# Supplementary figures and images for: Alternative polyadenylation factor CPSF6 regulates temperature compensation of the mammalian circadian clock
Source: PLoS Biol. 2023 Jun 28;21(6):e3002164. doi: 10.1371/journal.pbio.3002164 (PMC10335657; doi:10.1371/journal.pbio.3002164)

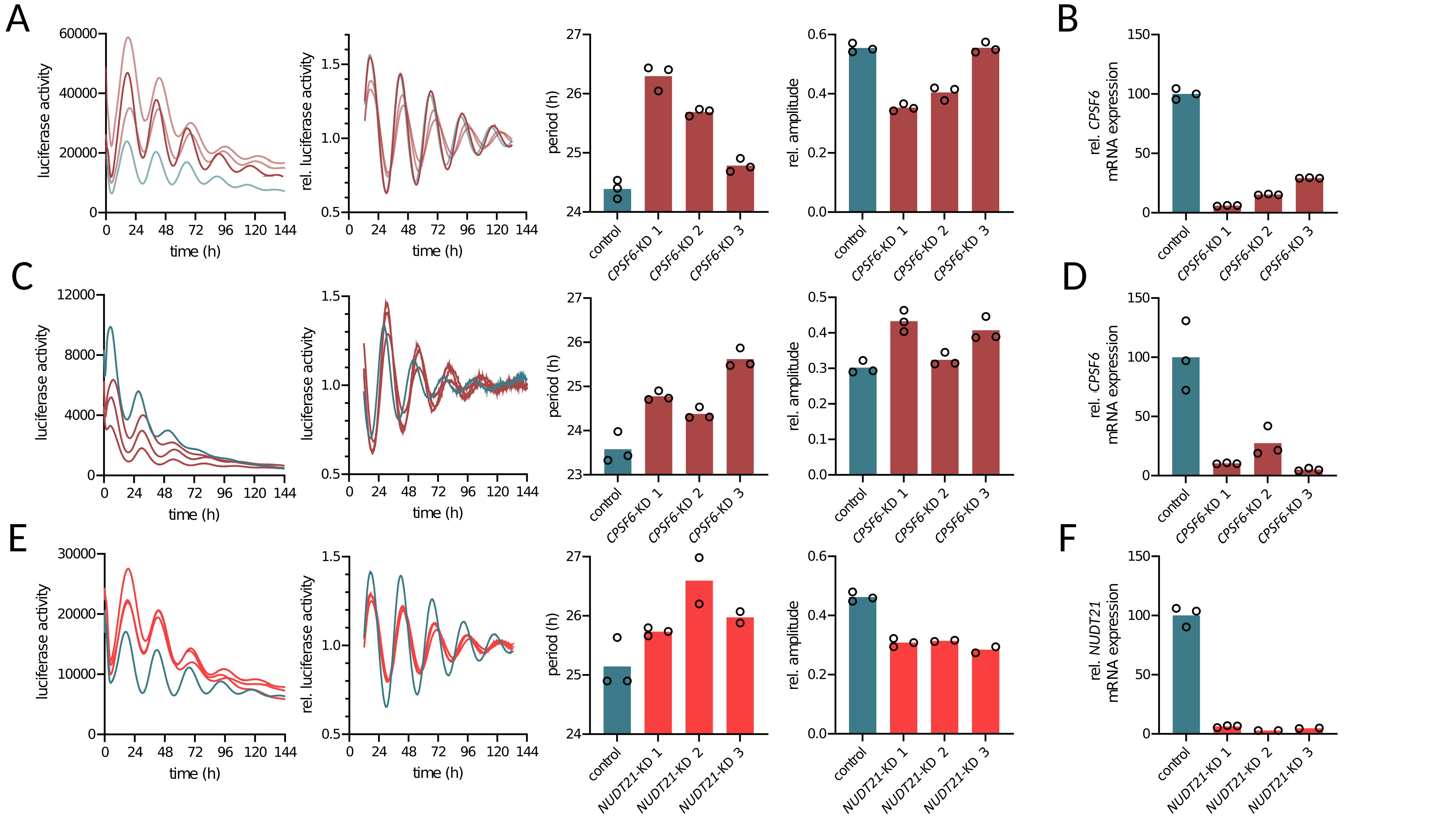

Supplement: S1 Fig — (A) Raw (first column) and detrended (second column) Bmal1-luciferase expression in human control (blue) and CPSF6-depleted (red) U-2 OS cells, using 3 different shRNA constructs. Corresponding periods and relative amplitudes as determined by the ChronoStar software are depicted in the third and fourth columns, respectively. Comparing the relative CPSF6 mRNA expression (B) with the corresponding periods (A, third column) reveals a dose-dependent (i.e., knockdown efficiency–dependent) lengthening of the free-running period in U-2 OS upon shRNA-mediated CPSF6 depletion. (C) Raw (first column) and detrended (second column) Bmal1-luciferase expression in mouse control (blue) and CPSF6-depleted (red) NIH3T3 cells, using 3 different shRNA constructs, together with the corresponding periods (third column) and relative amplitudes (fourth column). A comparison between the relative CPSF6 mRNA expression (D) and the corresponding period (C, third column) reveals a dose-dependent lengthening upon shRNA-mediated CPSF6 depletion in mouse NIH3T3 cells. (E) Raw (first column) and detrended (second column) Bmal1-luciferase expression in human control (blue) and NUDT21 (also known as CPSF5) depleted (red) U-2 OS cells, using 3 different shRNA constructs, together with the corresponding periods (third column) and relative amplitudes (fourth column). A comparison between the relative NUDT21 mRNA expression (F) and the corresponding period (E, third column) reveals a dose-dependent lengthening upon shRNA-mediated NUDT21 depletion in human U-2 OS cells. Raw data underlying panels A-F can be found in S2 and S5 Data. (TIFF) [file pbio.3002164.s001.tiff]

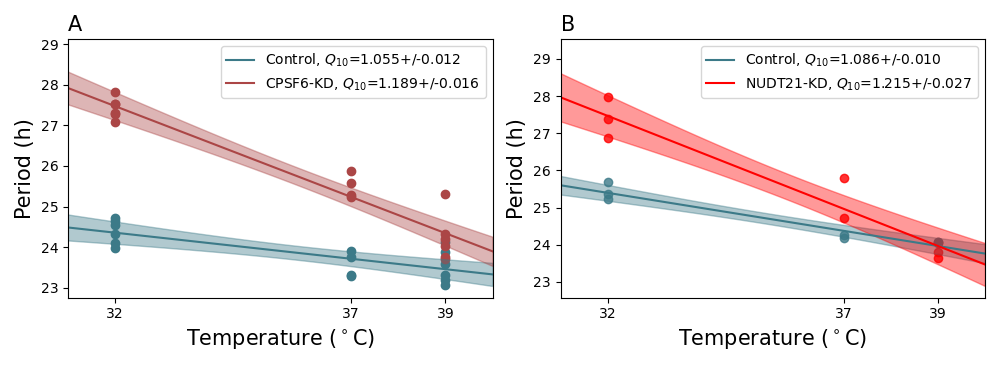

Supplement: S2 Fig — (A) Temperature response of circadian free-running period τ is statistically different (p<0.001) in wild-type and CPSF6 knockdown cells, assuming a linear dependency of free-running period τ on temperature T and testing against the null hypothesis that the 2 slopes, b1≈−0.13h°C and b2≈−0.45h°C, for wild-type (n1 = 16) and CPSF6 knockdown (n2 = 16) cells, respectively, are identical; see Materials and methods. Temperature coefficients have been obtained, using the equation Q10=(τ1τ2)10°C/7°C with τ1 and τ2 being the free-running period determined at 32°C and 39°C as obtained from the linear regression, respectively. Error propagation has been calculated via the Python uncertainties package. (B) Similarly, the temperature response of circadian free-running period τ is statistically significantly different (p≈0.001) in control (n1 = 7) and NUDT21 (n2 =8) depleted cells. Raw data and code underlying panels A and B can be found in S3 Dataset. (TIFF) [file pbio.3002164.s002.tiff]

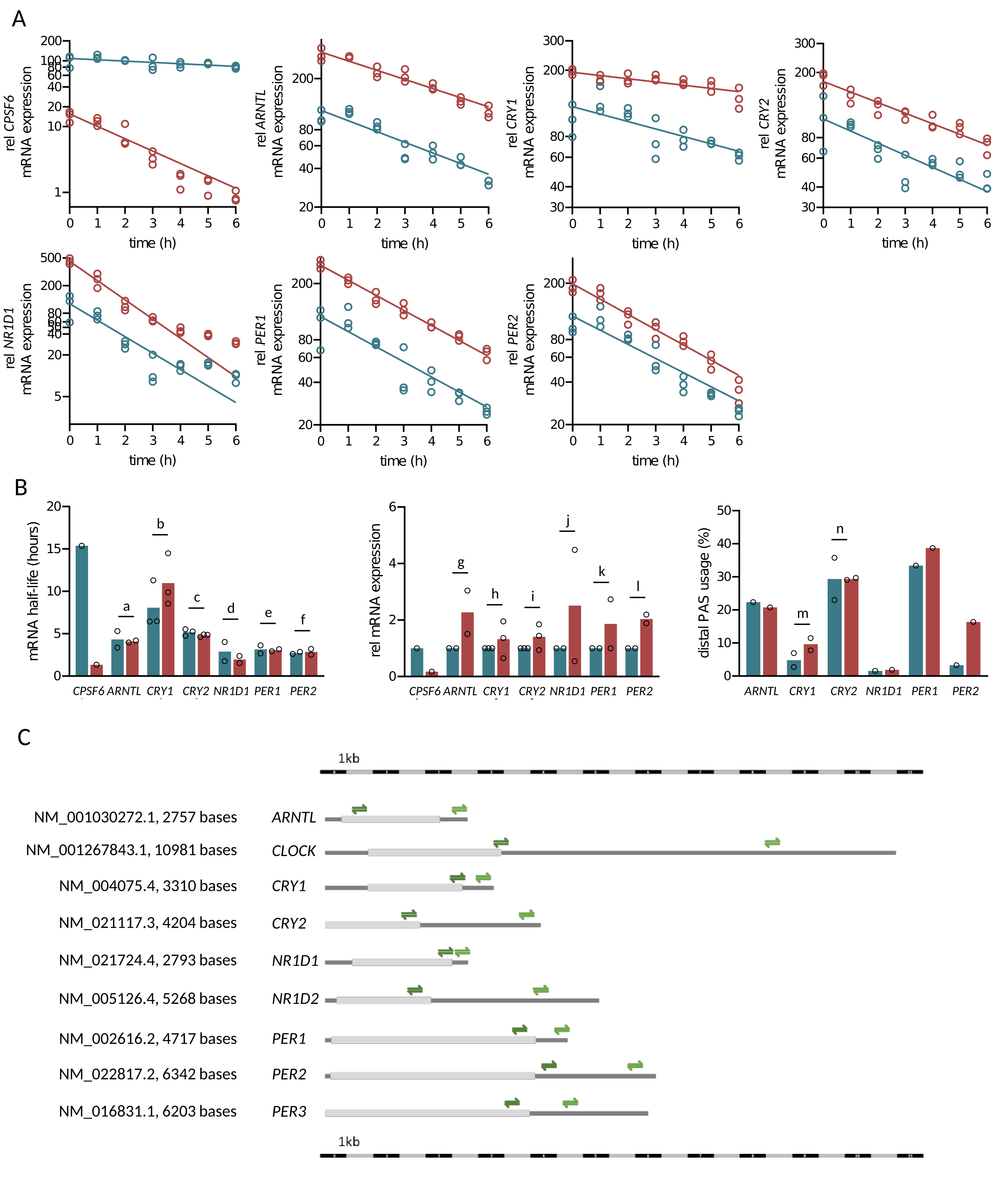

Supplement: S3 Fig — (A) Dynamics of CPSF6, ARNTL, CRY1, CRY2, NR1D1, PER1, and PER2 mRNA expression after application of the transcription inhibitor triptolide at time point 0 hours in wild-type (blue) and CPSF6-depleted (red) cells. Half-lives of the corresponding mRNAs are determined from the linear fits to the logarithmic data as depicted by the straight lines. (B) Bar plots, comparing the mRNA half-lives (left), total mRNA expression (middle), and distal polyadenylation site usage (right) in wild-type (blue) versus CPSF6-depleted cells. Each dot denotes results from an individual experiment where values of 3 technical replicates have been averaged. Bars denote the corresponding averages of these individual experiments. Results in (A) show dynamics of 3 technical replicates within 1 individual experiment in wild-type or CPSF6 knockdown cells, thus corresponding to 1 dot in the bottom panel. P values for a statistical comparison (t test) between the total mRNA half-life, total mRNA expression, and distal PAS usage between control and CPSF6-depleted cells are pa = 0.819, pb = 0.296, pc = 0.191, pd = 0.520, pe = 0.863, pf = 0.754, pg = 0.238, ph = 0.442, pi = 0.196, pj = 0.523, pk = 0.424, pl = 0.021, pm = 0.230, pn = 0.997. (C) Schematic representation of primer location, used to measure expression of total- (dark green) and long-3′ UTR (light green) isoform expression of the 9 core clock genes studied in Figs 1E, 1F, S3A and S3B. The distal polyadenylation site (PAS) usage is defined by the ratio of the long-3′ UTR expression (light green) divided by the total expression (dark green). While light gray regions depict the coding strand, dark gray regions depict 5′ and 3′ UTRs. NCBI Reference Sequence IDs, together with the corresponding length in base pairs (bp) and the gene symbol are given next to the schematic representation. Raw data underlying panels A and B can be found in S4 and S5 Data. (TIFF) [file pbio.3002164.s003.tiff]

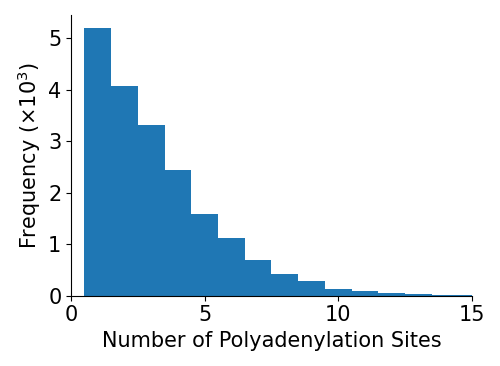

Supplement: S4 Fig — The RNAseH− approach detects alternative polyadenylation signals for more than 70% of the genes. Raw data underlying this figure can be found in S2 Table. (TIFF) [file pbio.3002164.s004.tiff]

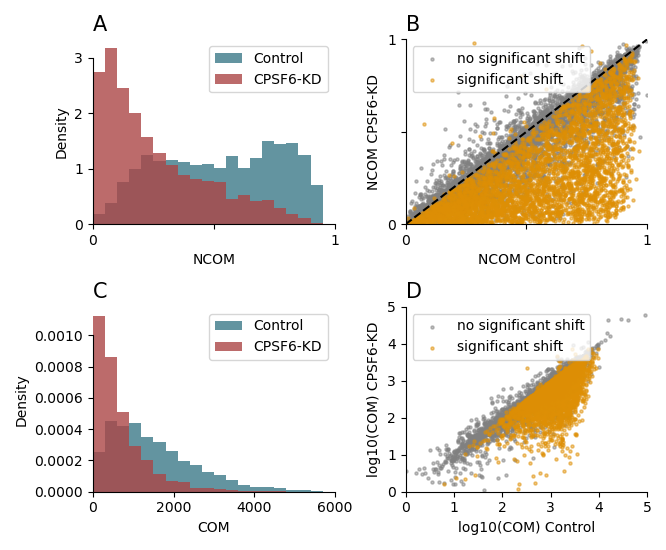

Supplement: S5 Fig — (A) Histogram of the center of read distribution along the 3′ UTR, normalized to the length of the isoform’s 3′ UTR (NCOM), in control (blue) and CPSF6-depleted (red) cells. Here, the distribution only considers those isoforms that show a significant shift in their NCOM upon CPSF6 knockdown. (B) Scatter plot of NCOM values in control versus CPSF6-depleted cells. Orange and gray dots depict isoforms exhibiting a significant or no significant shift in their NCOM value upon CPSF6 knockdown, respectively. (C) Histogram of the nonnormalized center of read distribution along the isoforms 3′ UTR (COM) in control (blue) and CPSF6-depleted (red) cells. (D) Decadic logarithm of COM values in control versus CPSF6-depleted cells. Again, orange and gray dots depict isoforms exhibiting a significant or no significant shift in their COM value upon CPSF6 knockdown, respectively. As in Fig 2B and 2C of the main text, all data are from experiments at 37°. Data underlying this figure are available from the NCBI Gene Expression Omnibus (GEO; http://www.ncbi.nlm.nih.gov/geo/) under accession number GSE185896 and is contained in S3 Table. (TIFF) [file pbio.3002164.s005.tiff]

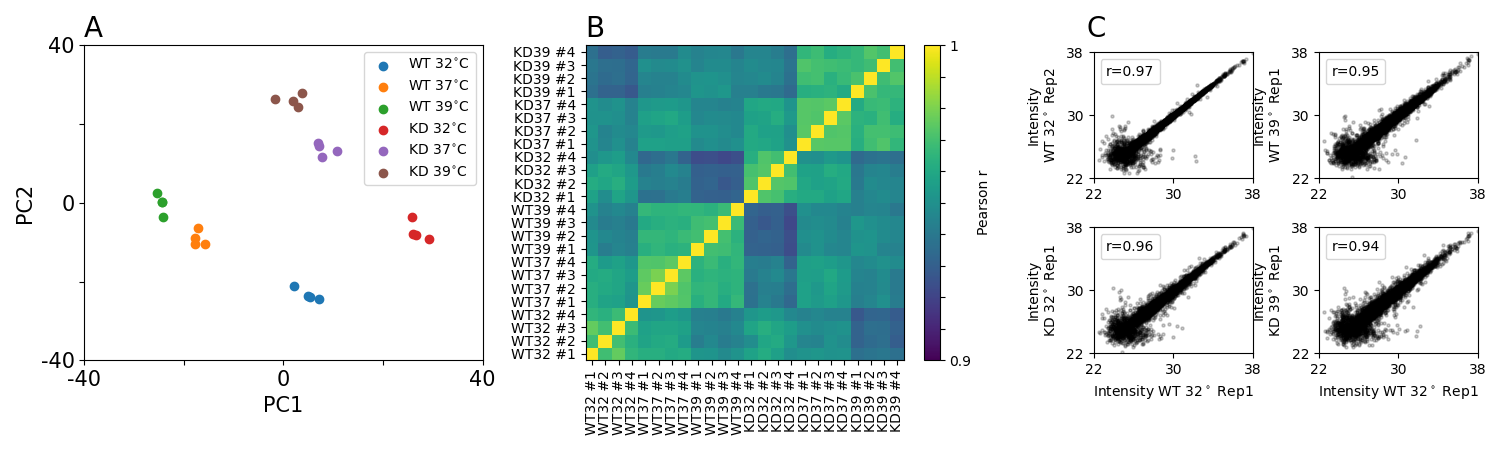

Supplement: S6 Fig — (A) Principal component analysis on log2-transformed protein LFQ intensities as determined by the MaxQuant software [99]. Technical replicates as indicated by equal coloring group together. (B) Correlogram of the Pearson correlation coefficients (r) from the log2-transformed protein LFQ intensities across measured samples. As expected, replicates with the same genetic background (wild-type versus CPSF6 knockdown) and at the same environmental temperature show the highest correlations of abundance values. (C) Representative scatter plots of log2 transformed protein LFQ intensities across different samples with the Pearson correlation coefficient (r). Data underlying this figure are available from the ProteomeXchange Consortium via the PRIDE partner repository (http://www.ebi.ac.uk/pride/archive/) with the dataset identifier PXD029343. (TIFF) [file pbio.3002164.s006.tiff]

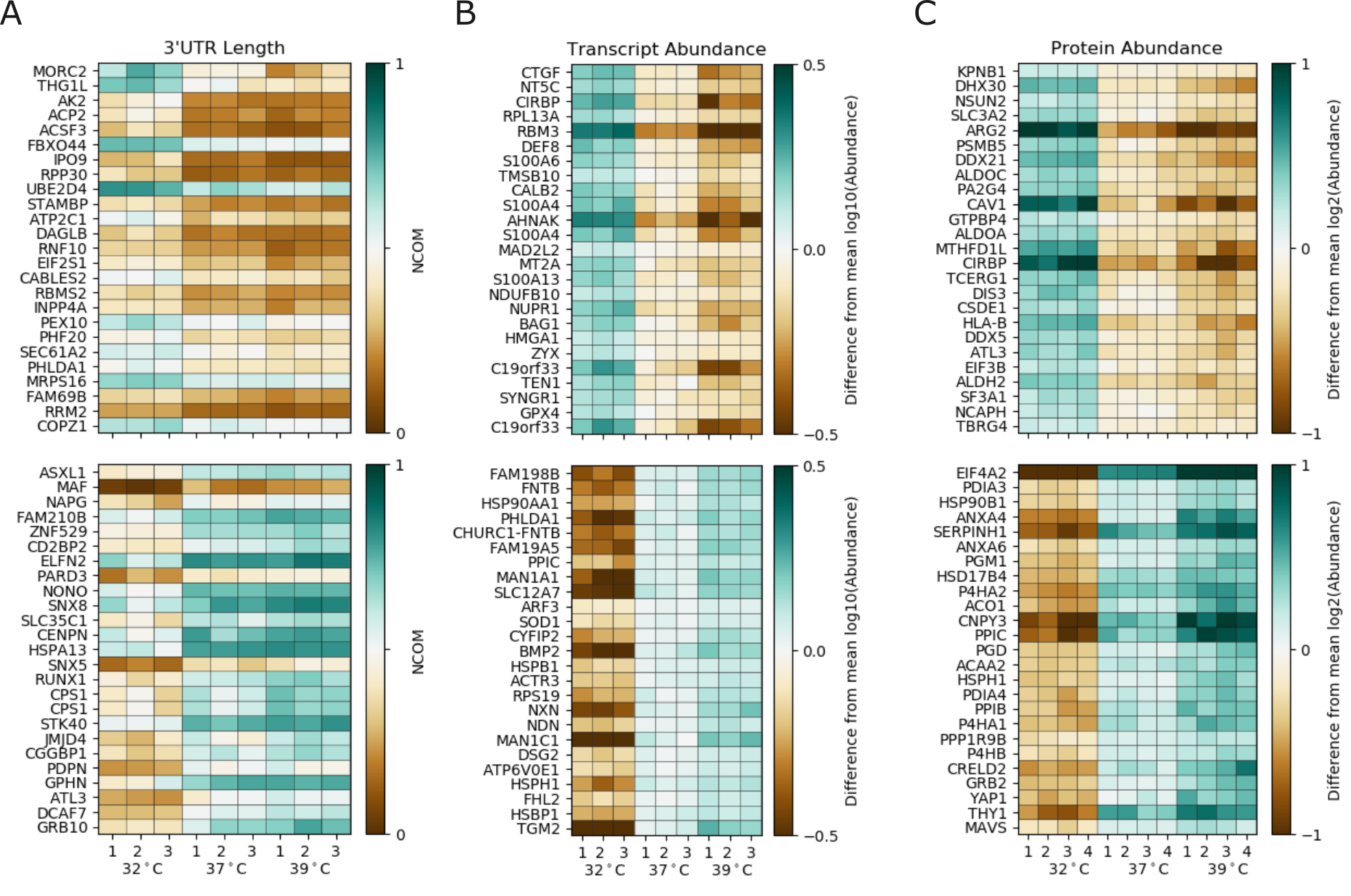

Supplement: S7 Fig — (A) Heatmap of 25 NCOM values that exhibit the most significant shortening (upper panel) or lengthening (bottom panel) of 3′ UTRs upon changes in environmental temperature in wild-type U-2 OS cells, sorted by increasing Benjamini–Hochberg corrected P values. All replicates at all 3 temperatures, namely 32°C, 37°C, and 39°C, are shown. (B) Same as panel (A), showing the 25 genes with the most significant down- (upper panel) or up-regulation (bottom) in gene expression upon increasing environmental temperatures. (C) Same as panels (A) and (B), showing the 25 genes with the most significant down- (upper panel) or up-regulation (bottom) in protein abundance upon increasing environmental temperatures. Data underlying this figure are available from the NCBI Gene Expression Omnibus (GEO; http://www.ncbi.nlm.nih.gov/geo/) under accession number GSE185896 and the ProteomeXchange Consortium via the PRIDE partner repository (http://www.ebi.ac.uk/pride/archive/) with the dataset identifier PXD029343 and are contained in S6–S8 Tables. (TIFF) [file pbio.3002164.s007.tiff]

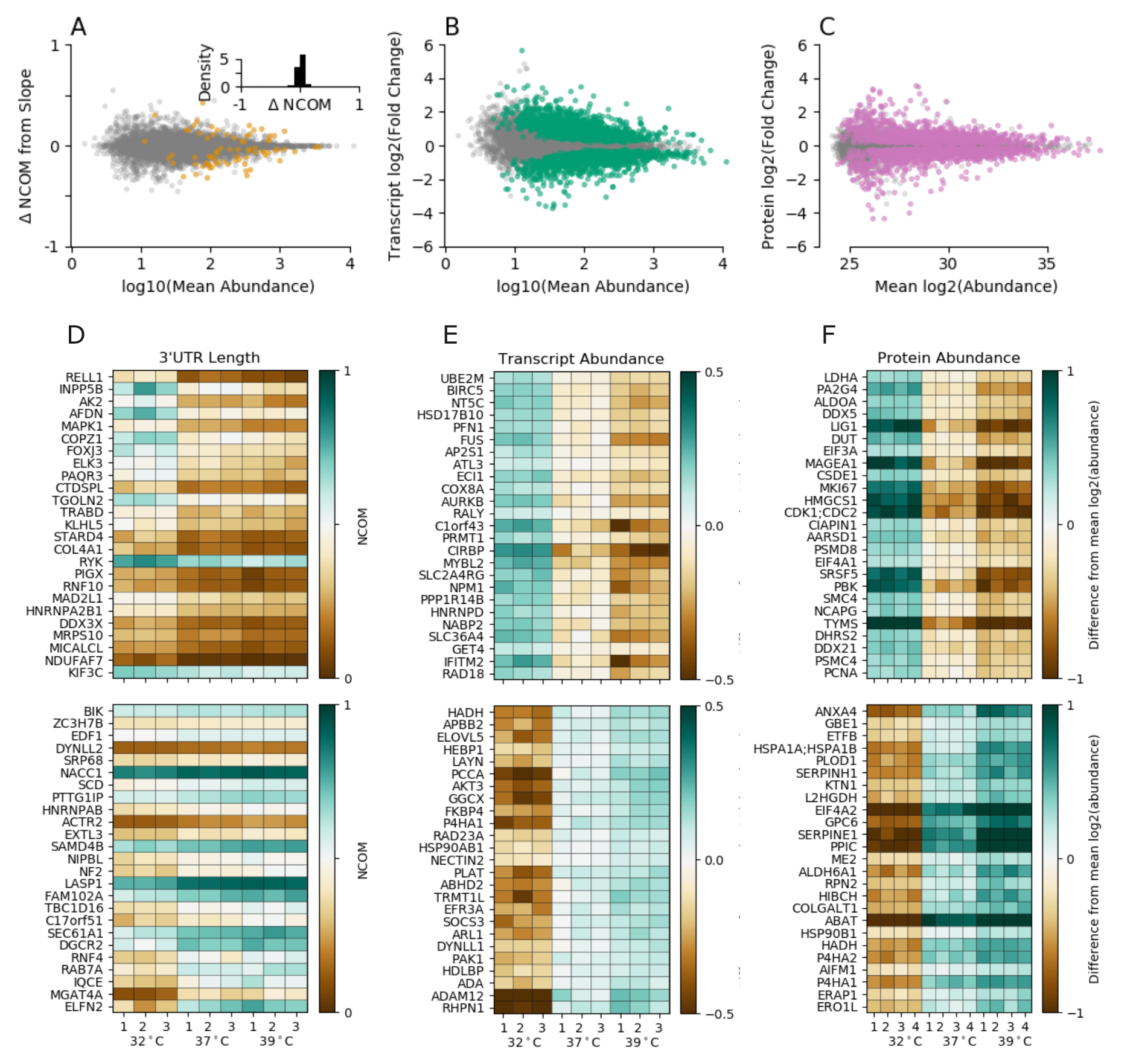

Supplement: S8 Fig — (A) MA plot showing the shift in 3′ UTR length upon temperature variations between 32°C and 39°C in CPSF6-depleted cells. Transcripts with a statistically significant change in 3′ UTR length at a 5% FDR (Benjamini–Hochberg corrected P values) are depicted by orange markers. No global shift in 3′ UTR length can be observed upon temperature alterations in CPSF6-depleted cells (inset). (B) Same as panel (A), showing the binary logarithm of transcript expression fold changes upon temperature variations between 32°C and 39°C on the ordinate. Transcripts with a statistically significant change in expression at a 5% FDR are depicted by green markers. (C) Same as panel (B), showing the binary logarithm of protein LFQ intensity fold changes upon temperature variations between 32°C and 39°C on the ordinate as well as the binary logarithm of the mean among all temperatures from the corresponding proteins. Proteins with a statistically significant change in abundance at a 5% FDR are depicted by pink markers. (D) Heatmap of 25 NCOM values that exhibit the most significant shortening (upper panel) or lengthening (bottom panel) of 3′ UTRs upon changes in environmental temperature in CPSF6-depleted U-2 OS cells, sorted by increasing Benjamini–Hochberg corrected P values. All replicates at all 3 temperatures, namely 32°C, 37°C and 39°C, are shown. (E) Same as panel (D), showing the 25 genes with the most significant down- (upper panel) or up-regulation (bottom) in gene expression upon increasing environmental temperatures. (F) Same as panels (D) and (E), showing the 25 genes with the most significant down- (upper panel) or up-regulation (bottom) in protein abundance upon increasing environmental temperatures. Data underlying this figure are available from the NCBI Gene Expression Omnibus (GEO; http://www.ncbi.nlm.nih.gov/geo/) under accession number GSE185896 and the ProteomeXchange Consortium via the PRIDE partner repository (http://www.ebi.ac.uk/pride/archive/) with the dataset id [file pbio.3002164.s008.tiff]

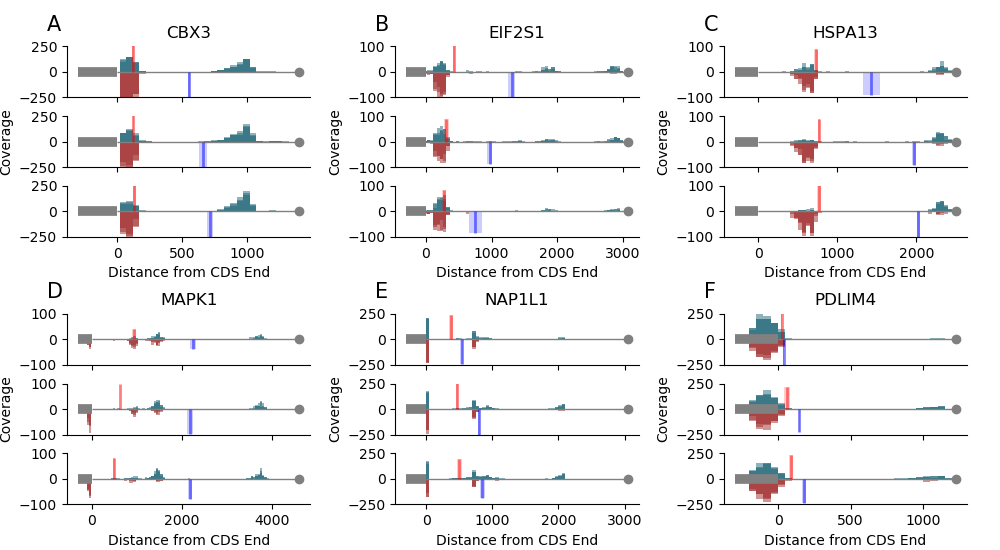

Supplement: S9 Fig — 3′ UTR read distributions (bars) and corresponding NCOM mean values (vertical bold lines) with standard deviations (shaded areas) of all 6 genes that show a differential temperature response at the level of 3′ UTR length, transcript expression, as well as protein abundance, shown for wild type (blue) and CPSF6 knockdown cells (red) at 3 different temperatures. The n = 3 technical replicates per condition are overdubbed in the bar plots. Data underlying this figure are available from the NCBI Gene Expression Omnibus (GEO; http://www.ncbi.nlm.nih.gov/geo/) under accession number GSE185896. (TIFF) [file pbio.3002164.s009.tiff]

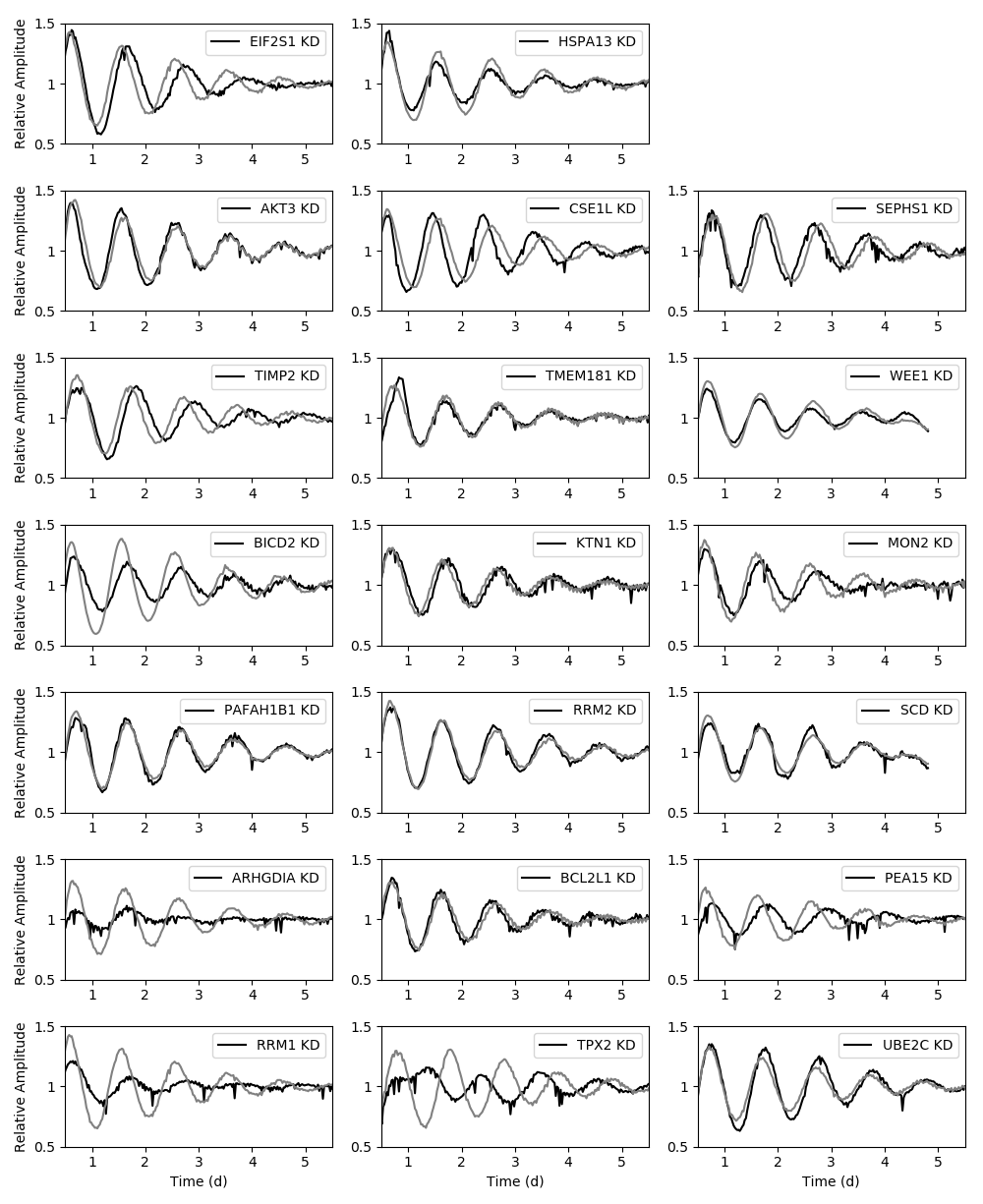

Supplement: S10 Fig — Relative amplitude of Bmal1-luciferase oscillations for RNAi constructs (black) and the corresponding control (plate mean; gray). Depicted are examples for genes showing a significant period shortening or lengthening in the RNAi screen as well as a differential temperature response at the NCOM, transript, and protein level (first row), the NCOM and transcript level (second and third row), the NCOM and protein level (fourth and fifth row), or the transcript and protein level (sixth and seventh row); compare Fig 5D. (TIFF) [file pbio.3002164.s010.tiff]

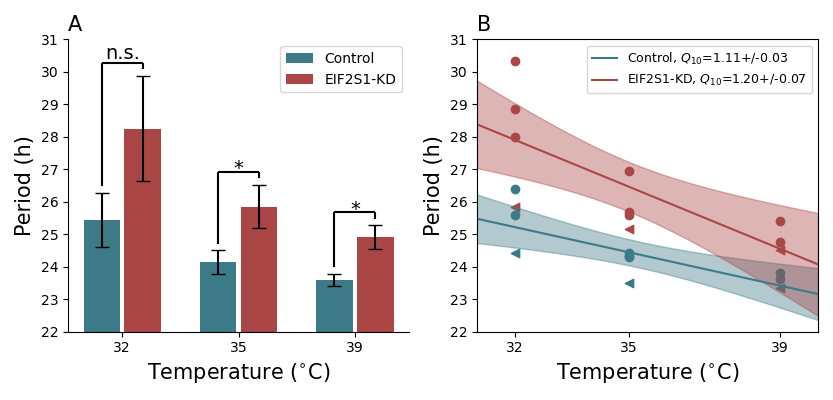

Supplement: S11 Fig — (A) Oscillatory periods of wild-type (blue) and EIF2S1 knockdown cells (red) are determined at 3 different temperatures as described in Materials and methods. While differences between control and EIF2S1-depleted cells are significantly different at 35°C and 39°C (p<0.01, t test), differences are slightly nonsignificant at 32°C (p≈0.068, t test) even though showing the same systematic period lengthening in knockdown cells. (B) As for the period difference at 32°C, even though linear relationships describing the temperature response of the circadian free-running period are not significantly different in control and EIF2S1-depleted cells (p⪅0.2), these data are consistent with the altered temperature compensation phenotype observed in CPSF6-depleted cells. Here, straight lines denote linear regressions as used to determine the corresponding period coefficient (Q10). Circle markers denote experiments exerted by using a TopCount luminometer, while triangle markers denote experiments exerted in light-tight single photomultiplier tubes (LumiBoxes). Data underlying this figure are available from S12 Table. (TIFF) [file pbio.3002164.s011.tiff]

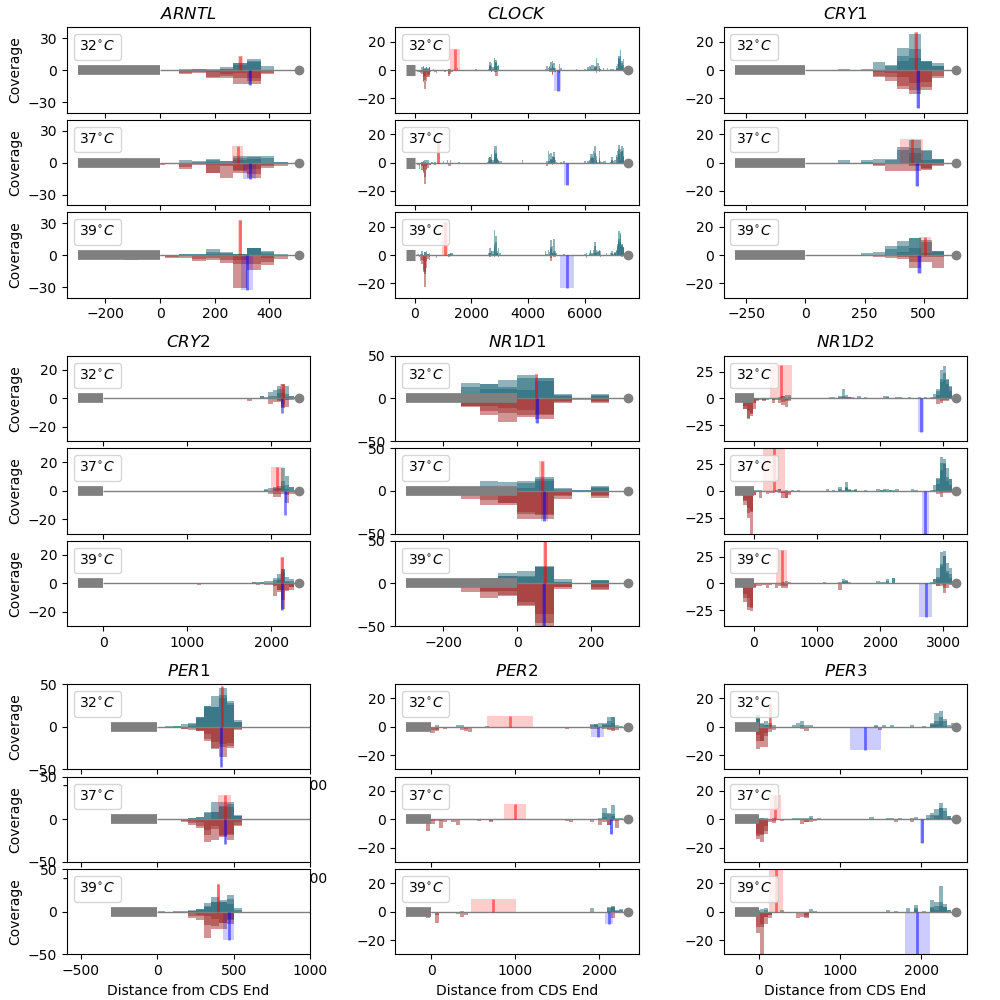

Supplement: S12 Fig — 3′ UTR read distributions (bars) and corresponding NCOM mean values (vertical bold lines) with standard deviations (shaded areas) of all 9 canonical clock genes investigated in Figs 1 and S3 for wild-type (blue) and CPSF6 knockdown cells (red) at 3 different temperatures. The n = 3 technical replicates per condition are overdubbed in the bar plots. Data underlying this figure are available from the NCBI Gene Expression Omnibus (GEO; http://www.ncbi.nlm.nih.gov/geo/) under accession number GSE185896. (TIFF) [file pbio.3002164.s012.tiff]

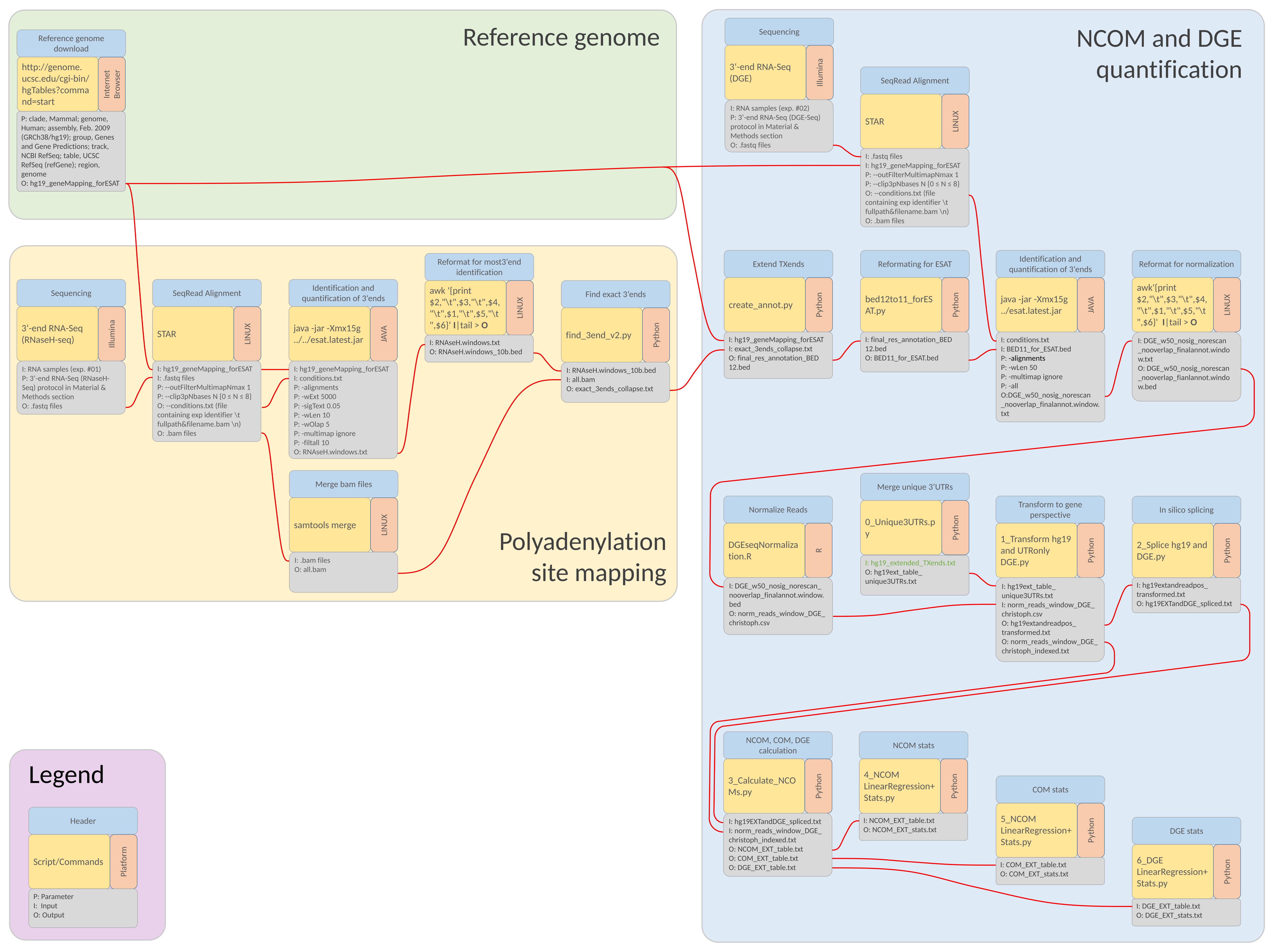

Supplement: S13 Fig — (TIFF) [file pbio.3002164.s013.tiff]

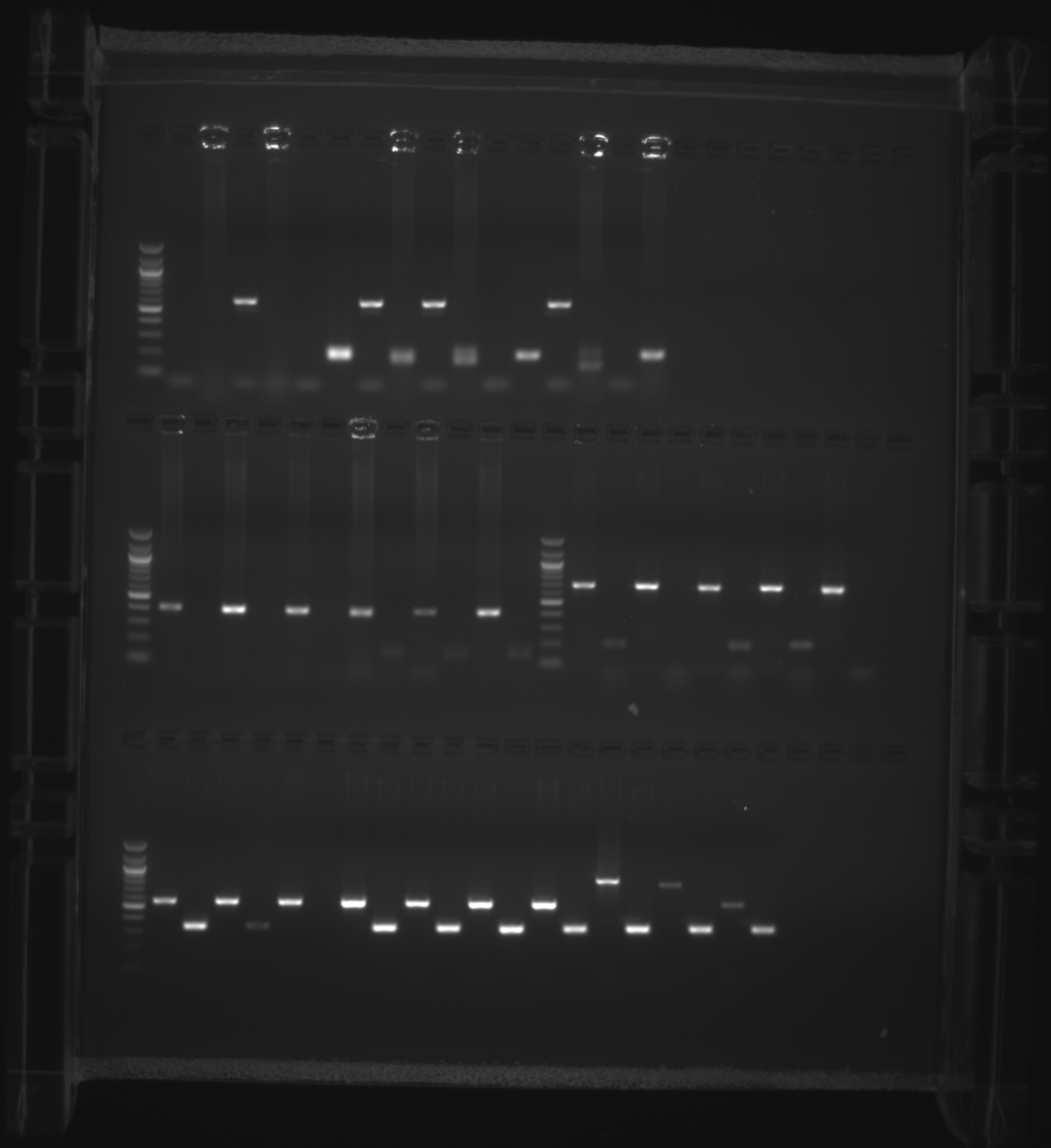

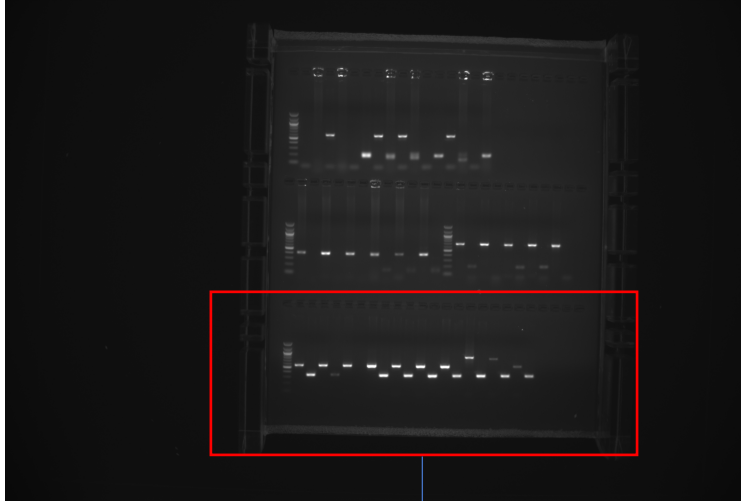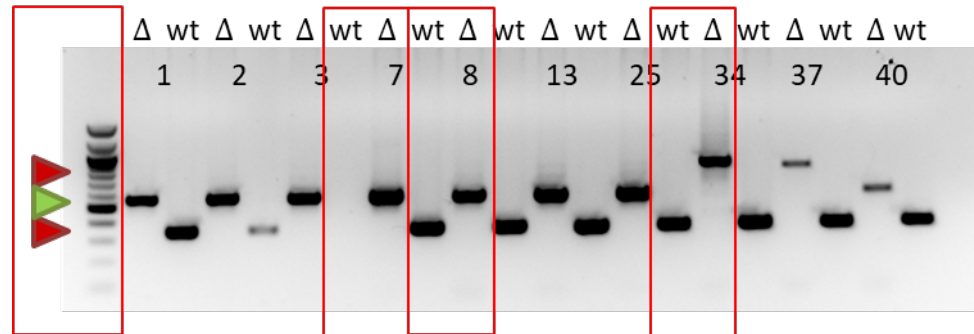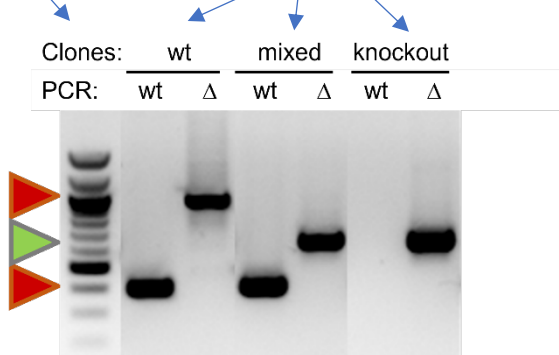

Supplement: S1 Raw Images — (PDF) [file pbio.3002164.s032.pdf]
