# Supplementary material for: Alternative polyadenylation factor CPSF6 regulates temperature compensation of the mammalian circadian clock
Source: PLoS Biol. 2023 Jun 28;21(6):e3002164. doi: 10.1371/journal.pbio.3002164 (PMC10335657; doi:10.1371/journal.pbio.3002164)
Supplement: S1 Text — (PDF) [file pbio.3002164.s026.pdf]

CRISPR-Cas9 mediated depletion of *CLOCK* 3' UTR polyadenylation sites does not phenocopy the *CPSF6* knockdown period lengthening phenotype

The *CLOCK* gene is a central player in the core clock machinery and has multiple alternative polyadenylation sites within a 7.5 kb stretch coding for the 3' UTR. We have shown that *CPSF6* knockdown leads to increased usage of more proximal alternative polyadenylation sites of the 3' UTR of *CLOCK* mRNA (Fig. 1C). Thus, we stated the hypothesis that the increased usage of more proximal alternative polyadenylation sites of *CLOCK* 3' UTR is directly or indirectly connected to the period lengthening in *CPSF6* knockdown cells. To test this, we sought to deplete either single polyadenylation sites of the *CLOCK* gene 3' UTR or an entire stretch of the *CLOCK* 3' UTR distal from the most proximal polyadenylation site in U-2 OS cells using CRISPR-Cas9 technology (supplementary text figure 1).

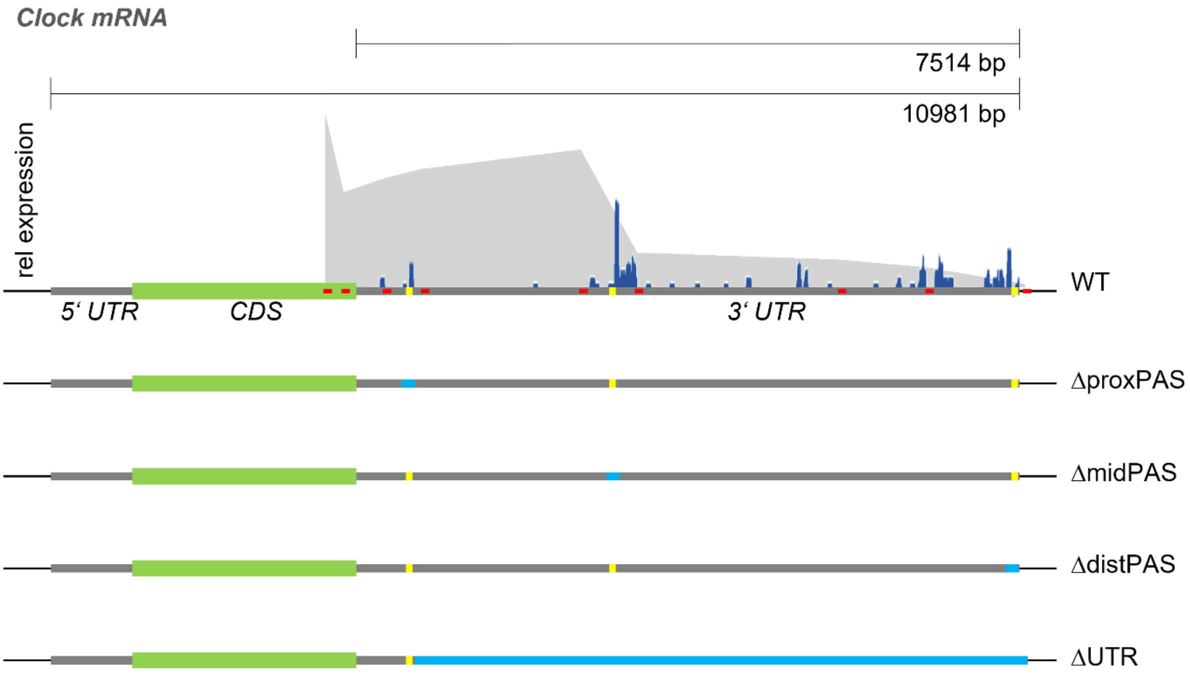

Supplementary text figure 1 schematically shows the almost 11 kb strech of *CLOCK* mRNA and its 7.5 kb of 3' UTR (WT). Using CRISPR-Cas9 with dual sgRNAs we depleted various regions (light blue bars - ΔproxPAS, ΔmidPAS, ΔdistPAS) in the clock gene coding for alternative polyadenylation sites (yellow bars, as confirmed by 3'end RNAseq results shown in dark blue histogram) of its 3' UTR or an entire stretch of the clock 3' UTR distal from the most proximal polyadenylation site (ΔUTR). Several qPCR primer sets were designed to quantify the expression of the 3' UTR of *CLOCK* (red bars indicate amplicon regions). The light gray shaded area indicates expression level of the *CLOCK* 3' UTR in U-2 OS WT cells.

Four different U-2 OS BLH reporter cell lines were generated each lacking one of the following regions in its 3' UTR: The proximal polyadenylation site (ΔproxPAS), the mid polyadenylation site (ΔmidPAS), the distal polyadenylation site (ΔdistPAS) or the entire stretch distal from the proximal polyadenylation site (ΔUTR) using dual expression of sgRNA and CRISPR-Cas9 (Börding et al. (2019) *Frontiers in Physiology* 10:577). All cell lines were subcloned and tested for the presence of the *CLOCK* gene WT or polyadenylation site depleted alleles using PCR and PAGE (supplementary text figure 2).

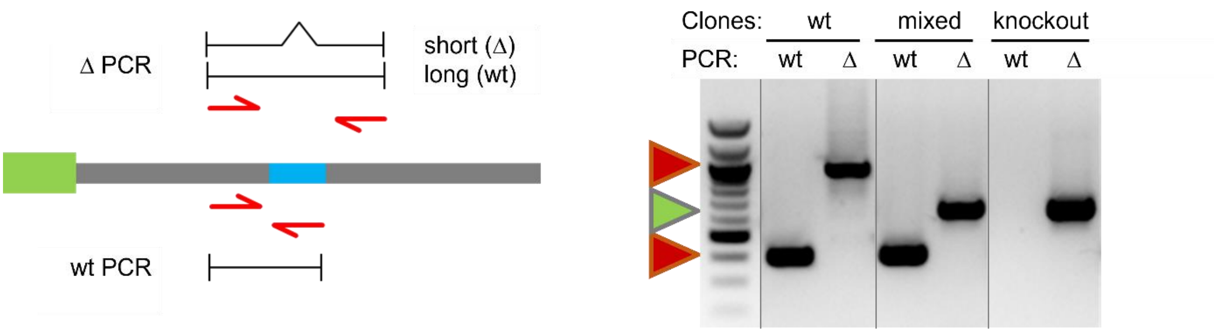

Supplementary text figure 2 schematically depicts the PCR primer design strategy (left panel) and exemplary PAGE results (right panel) to test for *CLOCK* ΔPAS genotypes in clonal U-2 OS BLH reporter cell lines. The successful depletion of the targeted *CLOCK* 3' UTR region was confirmed by i) presence of a shortened amplicon of the Δ PCR and ii) the absence of a WT amplicon in the WT PCR (most right lanes in PAGE panel).

A total number of 276 subclones have been derived from the various U-2 OS BLH *CLOCK* 3' UTR depletion cell lines (supplementary text table 1) using the limiting dilution method. While for some alternative polyadenylation site the success rates of depletion were in the range of our expectations (ΔproxPAS, ΔdistPAS and ΔUTR), we failed to identify clones in which all alleles showed a full depletion of the mid polyadenylation site. Interestingly, this polyadenylation site is most frequently utilized in U-2 OS BLH WT cells, as can be inferred from the *CLOCK* 3' UTR expression profile shown in supplementary text figure 1.

line	# of subclones	wt	mixed	knockout
WT	16	**10 (100%)**	0 (0%)	0 (0%)
ΔproxPAS	92	12 (13%)	70 (76%)	**10 (11%)**
ΔmidPAS	55	3 (5%)	52 (95%)	**0 (0%)**
ΔdistPAS	43	3 (7%)	23 (53%)	**17 (40%)**
ΔUTR	70	32 (46%)	24 (34%)	**4 (6%)**

Supplementary text table 1 summarizes the various *CLOCK* 3' UTR depletion cell lines and the numbers of subclones derived from U-2 OS BLH WT cells. Fully depleted (depletion on all alleles) number of clones and the success rate is shown in the last column.

Next, we determined the *CLOCK* 3'UTR expression profiles in U-2 OS BLH WT cell line and fully depleted ΔPAS subclones in *CPSF6* knockdown and control conditions, respectively (supplementary text figure 3). As expected, the WT cell line shows a massive reduction in expression of distal 3' UTR regions under *CPSF6* knockdown (red profile) compared to control (blue profile). In clones with full depletion of the most proximal polyadenylation site (ΔproxPAS) there was a slight increase of expression of 3' UTR regions up to the mid polyadenylation site under control and *CPSF6* knockdown conditions. Deletion of the distal polyadenylation site (ΔdistPAS) led to overall lower Clock mRNA expression, but rather unchanged 3' UTR expression profiles under both control and *CPSF6* knockdown conditions, respectively. Finally, full length depletion of the entire stretch of *CLOCK* 3' UTR downstream of the most proximal polyadenylation site (ΔUTR) further reduced overall *CLOCK* mRNA expression and resulted in background levels of expression profiles for the depleted region.

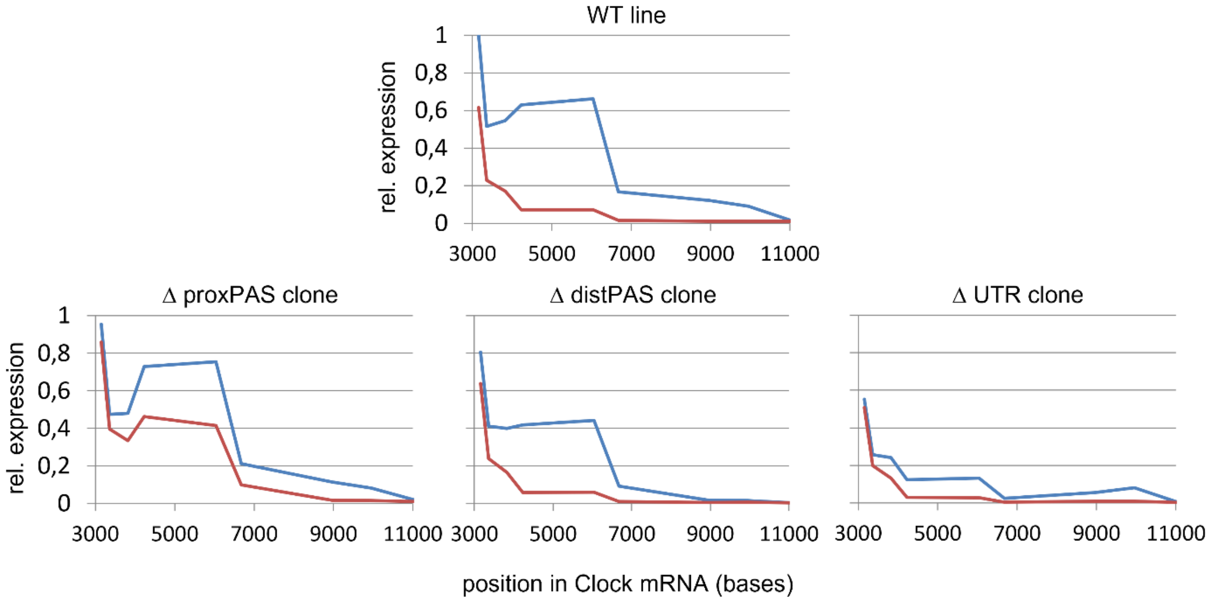

Supplementary text figure 3 *CLOCK* mRNA 3' UTR expression profiles in U-2 OS BLH WT cell line and subclones derived from CRISPR CAS targeted depletion variants (see SI figure 1 and SI table 1 for further details). Blue lines indicate *CLOCK* mRNA 3' UTR expression profiles in control conditions, whereas the red line mark expression profiles under *CPSF6* knockdown.

Finally, we tested whether depletion of polyadenylation site of *CLOCK* 3' UTR affects the period of the cell intrinsic circadian clock in U-2 OS BLH reporter cells using bioluminescence recording of dexamethasone synchronized cell cultures. We hypothesized that if *CLOCK* 3' UTR was directly or indirectly involved in mediating the *CPSF6* knockdown period phenotype, depletion of the polyadenylation sites downstream of the most proximal one (ΔUTR) would phenocopy the *CPSF6* knockdown even under control conditions, while *CPSF6* knockdown should not further lengthen the period in these subclones. However, the ΔUTR subclones tested did show normal wild-type behavior in respect to period upon control/*CPSF6* knockdown (supplementary text figure 4). Furthermore, depletion of the most proximal polyadenylation site of *CLOCK* 3' UTR did not result in normal wild-type periods upon CPSF6 knockdown, which would have been expected in case shortened *CLOCK* 3' UTR had mediated the *CPSF6* knockdown phenotype.

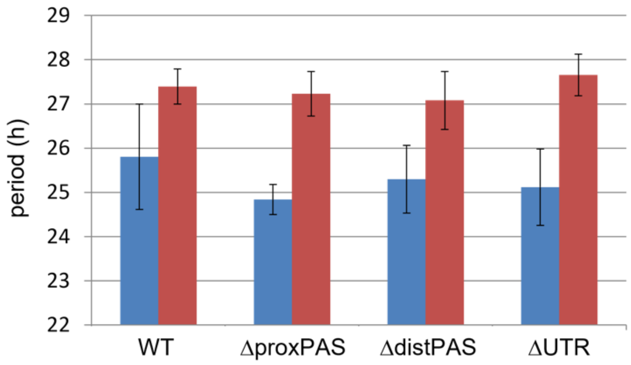

Supplementary text figure 4 Circadian clock period quantification in U-2 OS BLH WT reporter cell line and subclones derived from CRISPR CAS targeted depletion variants under either control (blue bars) or *CPSF6* knockdown (red bars) conditions. Shown are mean values +/- standard deviations of subclones tested (n=6-9 per genotype and knockdown condition).
