# Supplementary material for: Alternative polyadenylation factor CPSF6 regulates temperature compensation of the mammalian circadian clock
Source: PLoS Biol. 2023 Jun 28;21(6):e3002164. doi: 10.1371/journal.pbio.3002164 (PMC10335657; doi:10.1371/journal.pbio.3002164)
Supplement: Attachment — Submitted filename: Response_to_reviewers.pdf [file pbio.3002164.s033.pdf]

Response to Reviewers

Editor: The reviews are appended below. As you will see below, while the reviewers have commented that the data is of high quality and that the main conclusion of the study is interesting, they have also raised a number of important concerns which would need to be addressed before we could consider your manuscript for publication at PLOS Biology. The reviewers have highlighted the need for additional validation and identified potential issues with the experimental design and data interpretations which undermine the strength of the conclusions. Moreover, Reviewer 2 has commented that the study lacks deep mechanistic insights.

After careful discussion with the Academic Editor, we do not feel able to accept the manuscript in its current form, but would be willing to consider a revised version of your manuscript which provides stronger mechanistic insights and thoroughly addresses the reviewer concerns, with new data as needed.

We are grateful for the detailed and constructive comments of Reviewers 1-3. In the following step-by-step responses to all comments we have addressed each of the Reviewer’s concerns. We believe that the changes of the text, the additional computational analysis as well as new experimental data substantially improved our work.

Reviewer #1: In this manuscript, Schmal et al., examined the role of CPSF6 in regulating circadian temperature compensation, one of the defining characteristics of circadian rhythms. The molecular mechanisms underlying temperature compensation are indeed poorly understood and more studies in this area are definitely needed. The study includes a large quantity of data to shed light into this, however, the data presented in this manuscript do not support the authors' major conclusion and lack novelty. Some of the experimental design or data interpretation are not rational to this reviewer either. Please see the comments below for details. Because of these reasons, the reviewer cannot support the publication of this study.

We gratefully thank the reviewer for acknowledging the importance of our study and for the comments that we believe substantially improved the manuscript. In the following we address each of the reviewer’s concerns and have clarified our rational regarding the experimental design and data analysis.

Major points:

* The authors concluded that CPSF6 regulates temperature compensation based on Figure 1B, in which they knocked down CPSF6

and measured the period of Bmal1-luc in U2OS cells at three different temperatures. First, the authors need to test whether the efficiency of knock-down (i.e., the activities of Dicer, Argonaute, and RISC complex) and 3'-end processing are affected by the difference in temperatures to support their conclusion.

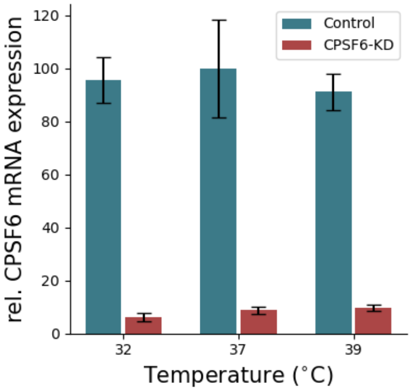

We gratefully thank the reviewer for this suggestion. Changes in the activities of Dicer, Argonaute or the RISC complex eventually translate into an altered knockdown efficiency. In order exclude that the observed temperature decompensation phenotype in U-2 OS cells is solely a consequence of a temperature-dependent *CPSF6* knockdown efficiency, we tested the expression of *CPSF6* in *CPSF6* depleted cells and non-silencing control cells at three different temperatures, see figure on the right. Here, the shRNA-construct corresponds to the construct labeled with "CPSF6-KD 1" in Fig. S1A,B. Indeed we find that the shRNA mediated *CPSF6* knockdown efficiency is resilient to temperature changes.

*   Second: Q10 values for non-temperature compensated biological reactions are generally in the range of 2.0-3.0 (Reyes et al., 2008). In contrast, the Q10 value reported for CPSF6 knock-down is 1.2 (Line 124, Fig. S2). Even if the difference in Q10 values between control and CPSF6 knock-down is statistically significant, this Q10 value is within the range of temperature compensation (0.85-1.2) as the authors mentioned (Lines 55-56 and 297-298). The subtle difference in Q10 could well be a statistical artefact without biological significance and it is far-fetched to conclude that CPSF6 knocked-down cells are resistant to temperature compensation, let alone "a tremendously impaired temperature compensation (line 322)".

We agree with the reviewer that the use of putatively over-excited adjectives or adverbs should be avoided in scientific literature. Along these lines we re-formulated our results more prosaically and exchanged corresponding expressions such as "[…]abolishes[…]" in the *Abstract* or the aforementioned "[…]tremendously impaired […]" by more careful expressions such as "significantly alters" or "significantly altered", respectively.

However, as temperature dependent chemical or biological reaction rate changes do not necessarily translate into period changes in a linear way (i.e. a 2-fold rate change does not imply a 2-fold period change) a Q10 value of 2-3 is not necessarily expected even for temperature de-compensated circadian clocks.

In order to access temperature-decompensation, we applied a careful statistical analysis for differential temperature responses between wild type and *CPSF6* knockdown cells: Instead of solely testing for significant period changes at a certain temperature (e.g. by a t-test), we specifically investigated if the temperature dependence is altered between control and *CPSF6* depleted cells by comparing whether the slopes describing the temperature dependency significantly deviates between both cases. By this means, we did show that *CPSF6* knockdown leads to a significant change in the temperature compensation behavior. In addition, knockdown of *CPSF6* leads to a significant ($p<0.01$, t-test at 32°C) period lengthening phenotype at lower temperatures with an approximately 3h longer period at 32°C, which is within the same order of magnitude than period phenotypes associated with knockdown of core clock genes such as e.g. the *period* gene in *Drosophila* (4-5h, Konopka and Benzer (1971) PNAS 68(9)). This emphasizes the potential biological importance of the found results.

In addition, the corresponding change of Q10 values between control and *CPSF6* knockdown cells from Q10=1.06 and Q10=1.19, respectively, is of the same order of magnitude as published in other studies reporting temperature de-compensated circadian clock phenotypes. To give an example we re-analyzed data of *Huang, Curtin and Rosbash (Science 267(5201):1169-1172, 1995)* that reports a temperature de-compensation phenotype in *Drosophila melanogaster* for flies that contain mutations in the dimerization domain (PAS) of the core clock protein *per*, see below.

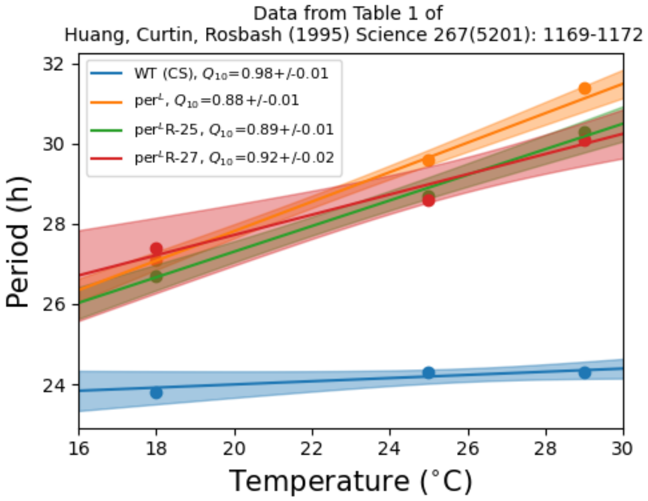

While the wild type *Canton* strain (CS) in *D. melanogaster* shows a slightly better temperature compensation (Q10=0.98) compared to our mammalian U-2 OS line (Q10=1.06) the increase in temperature sensitivity is of comparable magnitude in *Drosophila* per^L mutants and U-2 OS *CPSF6*-knockdown cells: While the former shows a decrease of the Q10 value from 0.98 (CS) to 0.88 (per^L), which translates into a change of the period by 1.6% and 13.2% from 20°C to 30°C in the CS and per^L flies, respectively, above described change in Q10 values translates into a change of the period by 5.5% and 18.7% from 30°C to 40°C in in *CPSF6* depleted U-2 OS cells. In the revised version of the manuscript, we substantially extended the section *Introduction* in order to introduce previously described temperature decompensation phenotypes and corresponding Q10 values among other organisms.

Finally, the importance of the role of *CPSF6* in buffering the circadian clock against external "perturbations" is further underlined by the findings of Kelliher, Stevenson, Loros and Dunlap (PLoS Biol 21(1): e3001961, 2023) that have been recently published in parallel while we have been working on this revision. Therein, the authors identified *CPSF6* to play a role in the nutritional compensation of circadian period in *Neurospora crassa* and mammals. Their findings in combination with our own data are consistent with the original proposition of Pittendrigh and Caldarola (PNAS 70(9): 2697-2701, 1973) that temperature compensation may be a special case of a more general mechanism that buffers the circadian free-running period against a larger set of unpredictable changes the circadian clock is likely to encounter in face of omnipresent intrinsic and extrinsic environmental fluctuations. *CPSF6* might be an important regulator of such overarching buffering mechanism that is conserved among different organisms such as humans and *Neurospora crassa*. In the revised version of our manuscript, we substantially expanded the last paragraph of the *Discussion* to embed these novel findings and their implications with our own data and added the corresponding pertinent references, i.e. (Kelliher et al. (2023) *PLoS Biol* 21(1): e3001961) and (Pittendrigh and Caldarola (1973) *PNAS* 70(9): 2697-2701).

* Similarly, the Q10 value for EIF2S1 is also 1.2 and within the range of temperature compensation (Fig. S10), although statistical analysis was not performed for this dataset.

As discussed above for *CPSF6*, the change in Q10 value and hence the alteration in the temperature sensitivity of the free-running period for *EIF2S1* depleted cells is comparable to alterations previously discussed for other temperature de-compensation phenotypes. Here, the increase of the Q10 value in *EIF2S1* depleted cells from 1.11 to 1.20 translates into a change of period by 11.1% and 20% from 30°C to 40°C in control and *EIF2S1*

depleted U-2 OS cells. Indeed, as described in the main text of our revised manuscript the slopes describing the temperature dependency of the free-running period are not significantly different between control and *EIF2S1* depleted cells (see *left panel* of the figure below). However, this is partly due to our conservative strategy of pooling shRNA constructs within the RNAi screen. As described in *Materials and Methods* and can be seen in Table S 12, individual screens have been considered as biological replicates. This means that even individually measured circadian periods from different shRNA constructs have been averaged within one screen – even if these are from different knockdown constructs (OligoIDs). A less stringent pooling criteria where all individual time series are individually considered would lead to a high significance (see *right panel* of the figure below). In the revised version of the manuscript, we discuss the phenotype of *EIF2S1* depleted cells in more detail. See also our answer to *comment 1* of *reviewer 2* for a discussion of additional experiments regarding the function of *EIF2S1*.

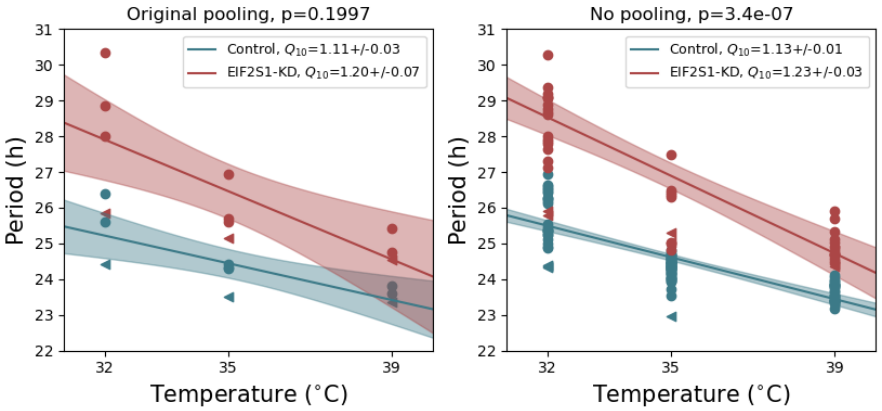

* It is unclear why the authors first screened siRNAs that would have different period or amplitude at 37C (Fig. S1) to understand the molecular mechanisms of temperature compensation. It would make more sense to test it at a different temperature. Please provide rationales for this.

At the very beginning of our study, we aim at searching for genes that could contribute to the temperature compensation of the circadian free-running period (tau). The reviewer is right that the most complete approach would have been to set up a whole genome screen at different temperatures in order to find all genes whose knock-down leads to an altered temperature compensation. However, we used two assumptions to simplify the analysis: 1) Temperature compensation is defined as the relative resilience of tau with respect to (adiabatic) temperature changes within the environment, resulting in a horizontal line in the period – temperature diagram with a slope close to zero, see blue line in the sketch below. In case of a temperature decompensated

clock, we expect a significant alteration of this slope as depicted by the red line in the sketch. Since we expect a period phenotype for all temperatures except those in the close vicinity of the point where the blue and red line cross (gray dot), we reduce the complexity of our initial screen by searching for period phenotypes at a single temperature and choose the most significant hit (i.e. CPSF6) for a further analysis of its temperature compensation phenotype. Along these lines, we accepted the risk of either false negative hits that lie close to the crossing point of both lines (gray dot) or false positive hits that may have a period phenotype while not showing temperature compensation, corresponding to the orange line in the sketch. 2) As a second simplifying assumption, we used prior knowledge from previous studies on temperature compensation that found post-transcriptional mechanisms to play a major role in temperature compensation and thus, we reduced the set of tested genes within our initial screen to those 1024 candidate genes annotated for posttranscriptional regulation and RNA processing withing the Gene Ontology (GO).

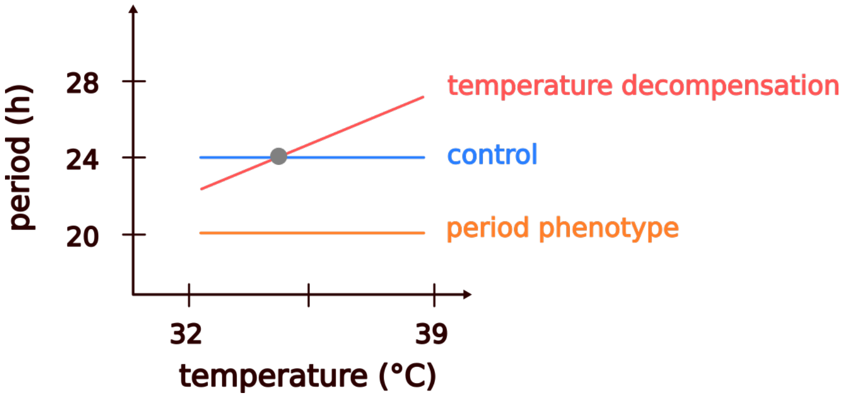

We substantially modified the first paragraph of section 2.1 in order to better describe our rational behind choosing a single temperature RNAi screen at the beginning of our study.

* Even if CPSF6 was the regulator for temperature compensation, it is unclear why the authors hypothesized that the mRNA stability of core clock genes would be the primary mechanisms to regulate temperature compensation. There are many biological consequences of alternative polyadenylation, not just changes in mRNA stability. Additionally, CPSF6 has a broad role in 3'-end processing, not just alternative polyadenylation.

We thank the reviewer for this comment, but we respectfully would like to ask to which additional roles in 3'-end processing the reviewer refers to. *CPSF6* is part of the polyadenylation machinery, consisting of more than 20 proteins. Within this machinery, two subunits of NUDT21 (or CPSF5) together with either one subunit of CPSF6 or one subunit of CPSF7 constitute the heterotetrameric complex cleavage factor I (CFI) that binds to UGUA cis-regulatory elements. It has been described that knockdown of *CPSF6* leads to a global change in polyadenylation

site selection and by this to overall shorter 3′ UTRs. Importantly, knockdown of *NUDT21* but not *CPSF7* phenocopies the knockdown of *CPSF6* (Li et al. (2015) *PLoS Genetics* 11(4):1-28). Similarly, we find that knockdown of *NUDT21* but strikingly not a knockdown of *CPSF7* phenocopies the effect of a knockdown of *CPSF6* on the circadian period in U-2 OS. In addition, knockdown of *NUDT21* phenocopies the effect of a knockdown of *CPSF6* on circadian temperature compensation. Taken together, we concluded that the effect of *CPSF6* knockdown on the circadian clock is likely due to its role within the polyadenylation machinery.

We have rewritten the first paragraph of Section 2.1 to better describe our line of reasoning. In addition, we added the new Supplementary Figure S2 B, showing how a knockdown of *NUDT21* phenocopies the effect of *CPSF6* knockdown on temperature compensation in U-2 OS.

Probably the best known and most studied consequence of alternative polyadenylation (APA) is its effect on microRNA (miRNA) function. Since (1) target sites for miRNA binding are generally located in 3′ UTRs (2) knockdown of *CPSF6* leads to a global 3′ UTR shortening and (3) miRNAs have been shown to modulate the stability and translation of their target mRNAs we believe that a very good educated guess for the mechanism of *CPSF6*-dependent regulation of clock gene expression would be by changes of clock gene stability via APA, even though other biological consequences of APA such as its effect on mRNA or protein localization have been described (Tian and Manley (2016), *Nat Rev Mol Cell Biol* 18(1):18-30).

* The authors should also briefly discuss what other mechanisms have been proposed to explain temperature compensation in mammalian cells (e.g., Beale et al., 2019, Shinohara et al., 2017, Isojima et al., 2009 etc)

We thank the reviewer for this suggestion. We substantially extended the corresponding paragraph of section *Introduction* to introduce proposed mechanisms underlying temperature compensation in mammalian cells. Along these lines, we added two new references, namely (Beale et al. (2019) J Biol Rhythms 34(2):144-153) and (Kon et al. (2021) Sci Adv 7(18):eabe8132).

* As the authors noted (Lines 182, 194-196, 210-211), there does not appear to be any novel findings from testing the effect of CPSF6 knock-down with APA, transcriptomic, and proteomic analyses performed at 37C.

After identifying *CPSF6* as an important regulator of temperature compensation of the mammalian circadian free-running period, we set out a multiomics analysis to uncover corresponding changes at

the 3′ UTR as well as transcript and protein expression level. The investigation of three different regulatory layers leads to a large and complex data set that, in our opinion, justifies a careful analysis at a constant temperature, prior to the subsequent more complex analysis of a differential temperature response between control and *CPSF6* depleted cells. In addition, to the best of our knowledge, this kind of analysis is novel for U-2 OS cells. Thus, we believe, the analysis at constant temperature is of interest by itself and the data set that we will make publicly available will be relevant to a broad readership interested in alternative polyadenylation (APA), especially in face of the previously reported tissue specificity of APA (Lianoglou et al. *Genes & Development* 27(21):2380-2396, 2013). Along these lines, at various points in the revised version of the manuscript, we more specifically set our results within U-2 OS cells into context with results from other tissues.

Minor points:
* Table S1 is incomprehensible. What does each column represent?

We revised Table S1, rephrased the column headings and substantially expanded the corresponding caption. In addition, Supplementary Table S1 now contains information about the number of shRNA constructs applied per gene as well as the corresponding nucleic acid sequences.

* Some figures are not mentioned in the text (e.g., Fig. 3D, 4D-F). The authors may want to remove these figures if they are not needed. The authors should also consider rearranging the order of figures to align with the main text. This would increase the readability of the manuscript and help future readers to better understand the study.

We gratefully thank the reviewer for pointing out this flaw. In the revised version of the manuscript, we carefully checked to have referenced all Supplementary Figures and Tables in the text.

Our figure arrangement made a compromise between thematic arrangement of the figures and references in the text. While almost all sub-figures are referenced in the same order as they appear in the figures there are a few exceptions in Figures 2 and 3 for visual purposes (as written in the first paragraph of *Section 2.2*) as well as a better thematic clustering of the figures. By this we ensure that Figure 2 deals with comparisons of control and *CPSF6* depleted cells at a single layer of regulation (i.e. 3′ UTR length as well as transcript and protein abundance) while Figure 3 deals with a comparison between these layers. In order to fully align the sub-figure ordering with the text, we would need to merge Figure 2 and 3 which we believe

deteriorates the visual appeal. However, if this is still requested by the reviewers or the editors, we are happy to rearrange the figures.

* Half-lives of mRNAs should be quantified to support the statement "we observed reduced mRNA half-lives (Fig. 1C)" (Line 139).

We quantified mRNA half-lives from linear fits to the logarithmic data (Fig. 1 C *middle*) and displayed results via bar plots (Fig. 1 C *lower left* panel). In order to emphasize this, we more carefully referenced the different sub-panels in the main text and shortly described in the caption of Fig. 2 that the quantified mRNA half-lives in the bar plots stem from the linear fits depicted in the *middle panel* of Fig. 2.

* Lines 116-117: please provide citations to support the statement that "thermosensitive regulation of pre-mRNA processing has been proposed as a mechanism underlying temperature compensation in other organisms“.

We added three references, namely Majercak et al. (1999) and Diernfellner et al. (2005, 2007) that discuss thermosensitive regulation of alternative splicing as a mechanism underlying temperature compensation and temperature sensation in *Drosophila melanogaster* and *Neurospora crassa*.

Reviewer #2: In the manuscript titled "Alternative polyadenylation factor CPSF6 regulates temperature compensation of the mammalian circadian clock", by Schmal et. al address the mechanisms of circadian temperature compensation. Here, authors showed that knock-down of CPSF6 both increases circadian period length and temperature decompensates the circadian clock. Multi-omic analysis upon knock-down of CPSF6 confirms the CPSF6 affects 3' UTR length, as well as transcript and protein abundance of a large number of genes. Finally, the authors have suggested that EIF2S1 is a key player in the CPSF6 knock-down cells that also play a key role in temperature decompensation.

The major contribution of the manuscript is identification of the alternative splice factor CPSF6 as a regulator of these two processes, and I think this point is made strongly. Mechanistic insights are not deep, however. The change in circadian period may well be due to increases in expression of many core genes. The change in temperature compensation is not well sorted out here; a number of candidates are proposed but not robustly validated, and no mechanistic insight is obtained.

The overall quality of the data is high, and the writing is clear (although see comments).

We gratefully thank the reviewer for acknowledging the quality of our data and writing. The manuscript improved by addressing all of the reviewers' concerns. Along these lines, we also discuss two additional experiments that have been carried out to gain more insights into how *CPSF6* together with *EIF2S1* affects the core clock machinery.

Major comments:

1. Though authors showed that knock-down of CPSF6 leads to circadian period length and temperature decompensation, the mechanism of action of CPSF6 in circadian temperature compensation remains largely elusive and not deeply addressed. In page 16 authors listed out 4 different ways to address the mechanism of action of CPSF6, but these questions were not addressed in the manuscript.

In order to further elaborate on the role of *CPSF6* together with *EIF2S1* on temperature compensation of the circadian clock, we extended our analysis along the 4 different ways to investigate the action of *CPSF6* as described in the manuscript. Along these lines, we revised the last paragraph of Section 2.4. First, we showed that *EIF2S1* knockdown leads to a period lengthening phenotype in the same order of magnitude as found for *CPSF6* depleted cells (see new Fig. S11 A). Second, even though not significant (due to a high standard deviation of the period data in *EIF2S1* knockdown cells at 32°C, see also our answer to *Comment 3* of *Reviewer 1*) our data is consistent with a temperature decompensation phenotype similar to that of *CPSF6-KD* cells.

Third, we further studied if an additional knockdown of *EIF2S1* in *CPSF6* depleted cells does lead to additive effects on the *CPSF6-KD* phenotype (see Figure *below*) in order to probe criterium 3, i.e., if a genetic depletion of *EIF2S1* in already *CPSF6*-depleted cells leads to an additional effect on the circadian period phenotype. First, it turns out that free-running periods of *EIF2S1* depleted cells at 35°C are similar to those of *CPSF6* knockdown cells, i.e. while a significant period difference can be detected between non-silencing control (WT_shNS) and *EIF2S1* knockdown cells (WT_shEIF2S1; $p=0.019$, t-test, $n=3$) there is no significant difference between *CPSF6* and *EIF2S1* depleted cells ($p>0.05$, t-test, $n=3$), see Fig. below A. Second, double knockdown of *CPSF6* and *EIF2S1* (shCPSF6_shEIF2S1) shows no additive effect in comparison to cells where only *CPSF6* (shCPSF6_shNS or shCPSF6_shCPSF6) is depleted ($p>0.05$, t-test, $n=3$), thus, being consistent with criterium 3 of the *Main text*. However, a qPCR-based validation experiment regarding the knockdown efficiency

(of one out of four shRNA constructs used in the screen depicted in panel A) shows that *EIF2S1* expression levels in shCPSF6_shEIF2S1 double-knockdown cells is reduced only to a level of about 84% of the control expression (see Fig. B below). Thus, the observed phenotype may be explained to a large extent by the initial *CPSF6* knockdown itself. Thus, even though being consistent with criterium 3 and hence with the hypothesis that *EIF2S1* may mediate the effect of *CPSF6* knockdown, we believe that the evidence is not strong enough to include these results in the *Main text* of the manuscript. We will follow up these experiments in future more mechanistic studies.

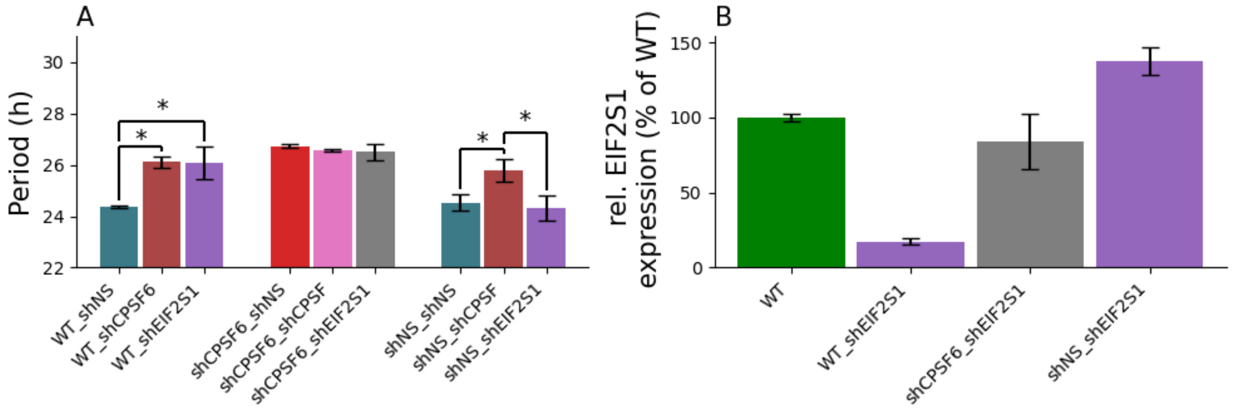

Experiments regarding the fourth criterium (i.e., if the impairment of polyadenylation signals within the 3' UTR of *EIF2S1* leads to a circadian phenotype similar to that of *CPSF6* depleted cells) are, due to limited resources, beyond the scope of this revision. Furthermore, the focus of this manuscript has been on identifying *CPSF6* as an important regulator of clock period and temperature compensation and the detailed multi-omics bioinformatic analysis to further reveal candidate genes underlying circadian temperature compensation. A further in depth characterization of the involved molecular mechanisms will be subject of future work.

2.a Authors identified EIF2S1 as a key player in temperature compensation from the CPSF6 knock-down cells using multi-omic approaches, but how these two molecules are connected in regulating circadian period and temperature compensation remains unknown and unaddressed.

In order to further investigate how these two molecules are functionally connected in regulating the circadian period phenotype and temperature compensation we carried out an epistasis experiment as described in the previous comment. Unfortunately, the *EIF2S1* expression in shCPSF6_shEIF2S1 double

knockout cells is not reduced to an extend comparable to the shEIF2S1 single knockout cells. Thus, it cannot be excluded that the lack of additional effect on circadian period compared to *CPSF6* single knockout cells is due to the rather inefficient knockout. Due to limited resources we have not been able to pursue this direction further, even though initial results were at least consistent with our hypothesis that *EIF2S1* mediates the impact of *CPSF6* knockout on the circadian period and temperature compensation phenotype.

2.b In fig. 5E second column, though there are differences in 3' UTR length and transcript of EIF2S1 in CPSF6 knock-down cells, the protein abundance remains almost the same in wild type and knock-down conditions. So, I think it is not clear whether EIF2S1 fulfill all the three criteria to be selected as a key player.

EIF2S1 is one of the 678 genes whose protein abundance shows a significantly different temperature response between control and *CPSF6* depleted cells. The enlarged plot below demonstrates that slopes describing the corresponding temperature response are indeed statistically significantly different:

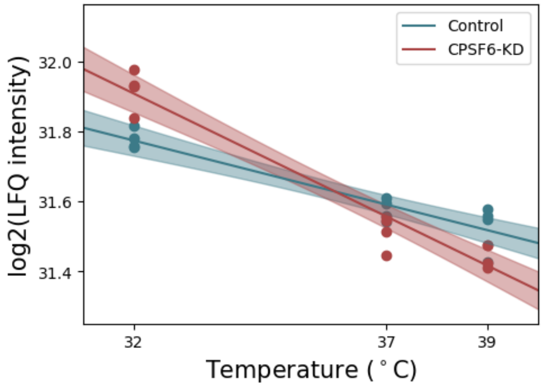

3. It has been mentioned in the manuscript that either balance hypothesis or critical reaction hypothesis are the popular models for temperature compensation based on the previous publications. But in the current study, results support neither of these two models nor the authors propose a third alternative mechanism to explain mechanisms of temperature compensation, especially how CPSF6 alters the core clock machinery in lower and higher temperature conditions to do temperature compensation.

We agree with the reviewer. We mentioned the balance or critical reaction hypothesis as part of an historical overview over the development of theories on temperature compensation. It was not our intention to suggest that we solve this longstanding problem by our study. Thus, we agree that it is inappropriate to discuss these issues in the introduction and transferred corresponding paragraphs from the *Abstract, Introduction* and *Results* sections

to the *Discussion*. While the critical reaction hypothesis assumes that critical period determining processes are temperature insensitive, the balance hypothesis assumes that different period-depending processes counterbalance each other. Thus, if these critical period determining processes occur within the regulatory layers investigated by our study one could, in principle, search for patterns that are in favor of one or the other hypothesis. For example, in EIF2S1 (as well as CBX3 and HSPA13 but not MAPK1 that behaves the other way around, see Fig. 5E) a temperature dependent 3′ UTR length coincides with largely temperature independent transcript and protein abundance while *CPSF6* knockdown leads to temperature insensitive short 3′ UTRs that now coincides with temperature dependent transcript and protein abundances (Fig. 5E).

In order to further study if and how *CPSF6* affects temperature compensation via the core clock machinery, for the revised version of the manuscript, we investigated how a CRISPR-Cas9 mediated depletion of the polyadenylation sites of the *CLOCK* 3′ UTR affects the circadian period phenotype in U-2 OS cells. Along these lines, we focused on the *CLOCK* gene because (1) it has the largest 3′ UTR among all canonical clock genes (2) *CLOCK* is essential for normal circadian behavior (see e.g. Vitaterna et al. (1994) Science 264: 719-725) (3) it shows a large shift in PAS usage in our analysis that is accompanied by reduced half-life and total mRNA expression (Fig. 1 C). Other clock genes apart from *NR1D2* with has a much shorter 3′ UTR compared to *CLOCK* do not show a systematic shift in 3′ UTR as well as alterations in mRNA half-life and expression (Fig. 1C and Fig. S3). However, as a result of our analysis we find that neither short or long isoforms mimic the phenotype of *CPSF6* knockdown nor do we find an indication for the role of *CLOCK* 3′UTR in regulating the circadian period length. We shortly summarize these results in the final paragraph of *Section* 2.1 as they are a further motivation to search for regulators of temperature compensation outside the canonical set of clock genes. In addition, we added Supplementary Text S1 to describe these results in further detail and added the reference (Vitaterna et al. (1994) Science 264: 719-725) to the manuscript.

Methodologic issues:
I believe that RNAseq can be quantitative and reproducible. I also believe (based on many experiences) that Mass spectrometry-based protein quantitation is MUCH less reliable than RNAseq. So, while I don't think it's important to validate RNAseq with qPCR, I do think it is critical to verify MS-identified changes in protein abundance using other methods, such as immunoblots.

We thank the reviewer for this comment, but we respectfully believe that this request could be considered currently outdated.

Technological, methodological, and computational advances in the last two decades have revolutionized the analysis and quantification of proteins and post-translational modifications using mass-spectrometry based, in particular label free quantitative proteomics (10.1016/j.molcel.2013.01.029). Nowadays this technique is highly reproducible, precise and sensitive in a degree that cannot be compared with immunoblot performance as many world-leading proteomics researches have been publicly expressing (10.1074/mcp.E113.031658, 10.1038/s41477-022-01314-8, 10.1021/pr800463v).
In the following points we expose the main reasons for which we consider mass spectrometry data superior to immunoblotting results.

- Quality of the analysis: Protein quantification/identification with immunoblotting is based on an antibody with a particular, not always highly accurate, specificity and affinity for the protein epitope. Even if specificity/affinity is high this can be easily altered by the presence of post-translational modifications in the epitope sequence of the protein. Mass spectrometry-based label-free proteomics yields protein identification based on several peptides per protein which makes wrong identifications improbable.

- Quality of the results: Protein quantification with immunoblotting is semiquantitative at its best, as it depends on the signal of a band in the blot. This signal can be specific or unspecific depending on the quality of the antibody, and even more can be altered by uncompleted transfer to the membrane. Moreover, the size/shape of the band and thus the accuracy of the quantification, can be also altered by the presence of post-translational modifications in the protein. As indicated above, mass spectrometry-based label-free quantification of a protein relays in the quantification of individual peptides. Thus, the availability of several independent data points for each peptide leads to a more accurate protein quantification.

- Performance features: Both techniques have features that determine their performance, such as reproducibility, limit of detection and linear dynamic range. Mass-spectrometry features undoubtedly outperforms those from the immunoblot.

Taken all this together we hope that we convince this reviewer that although immunoblotting offers a visual depiction of a singular protein, its limitations render it an incomplete validation for quantitative proteomic data.

A single RNAi against EIF2S1 is not adequate to draw conclusions. RNAi is rife with off-target effects. Studies that reply heavily on RNA interference (RNAi) to probe biological processes such as circadian rhythms must include appropriate controls such as those described in Nat Rev Drug Discov 9, 57-67 (2010). doi 10.1038/nrd3010. See specifically the paragraph 'Recognizing and confirming on-target effects.' Such controls must include use of multiple si/shRNAs for the same target, and/or (the best experiment) rescue of the phenotype by expression of target sequences refractory to si/shRNA.

We are sorry that we have not properly reported the details of our experimental setup in this case. Indeed, multiple individual shRNA constructs have been generally used in order to target a single gene. In case of *EIF2S1* a total of 6 and 4 different shRNA constructs have been used in the large- and small-scale RNAi screen, respectively. On average approximately 2.4 shRNA constructs have been used among the 1024 genes targeted in the large-scale RNAi screen, depending on the availability of constructs per gene from the supplier. We revised Table S1 to append all important information such as the number of shRNA constructs as well as their nucleotide sequences. In addition, we added a new Table S12 that contains information about the data and shRNA constructs underlying Supplementary Figure S11. We thank the reviewer for the reference regarding shRNA validation which we added to the corresponding paragraph in *Materials and Methods*.

Unfortunately, due to limited resources, the proposed rescue experiment could not have been done. However, due to the robust period lengthening phenotype in *EIF2S1* knockdown cells at three different temperatures (Fig. S11) we are confident about the validity and specificity of the found *EIF2S1* knockdown phenotype.

Minor comments:

For the initial RNAi screen, how many siRNA per gene were used?

We added this information as well as the corresponding nucleic acid sequences to Supplementary Table S1.

Table S1 (and others): there are many columns with cryptic headings. What do they mean? These should be defined. What is column 1? with no heading? What is "174"?

We have revised Table S1, removed unnecessary information, modified the table headers and substantially appended the corresponding caption. The revised version of Table S1 now contains information about the number of shRNAs per gene as well

as the corresponding nucleic acid sequences, see also our previous response.

Line 109: "CPSF6 is part of a multi-protein complex that is important for 3'-end processing of transcripts defining the polyadenylation site and thus the 3' UTR length." I wanted to learn more about CPSF6 and its known cellular functions. As a reader, I would have appreciated some references to papers about CPSF6.

We have substantially extended the last paragraph of Section *Introduction* in order to introduce the molecular interaction partners and cellular functions of *CPSF6* more thoroughly. A focus has been made on its role within the complex cleavage factor I CFI together with *NUDT21* and *CPSF7* and their role for polyadenylation site selection.

NUDT21 is stated to be a heterodimeric binding partner of CPSF6. This deserves a reference as well. I found several sources stating that CPSF6 is in a tetramer, not a heterodimer. Which is correct?

The reviewer is right. The polyadenylation complex cleavage factor I (CFI) is a heterotetramer composed of two CFIm25 subunits (CPSF5 or NUDT21) and either CFIm68 (CPSF6) or CFIm59 (CPSF7). Since the tetramer is composed of only two different kinds of subunits it is sometimes referred a heterodimer (see e.g. Tian and Manley (2016), *Nat Rev Mol Cell Biol* 18(1):18-30). However, we changed the text in order to account for the rather tetrameric structure of the complex. We appended the introduction and added corresponding references.

116: "thermosensitive regulation of pre-mRNA processing has been proposed as a mechanism underlying temperature compensation in other organisms" should be referenced.

We added corresponding references that discuss thermosensitive alternative splicing in *Drosophila* (Majercak et al. 1999) as well as *Neurospora* (Diernfeller et al. 2005 and 2007) and its relation to temperature compensation of the circadian clock.

Figure S3 and others: the figure legends would be easier to read if it didn't refer to a different figure legend. I suggest putting much more information into the figure legend.

We gratefully thank the reviewer for this suggestion. We substantially increased the information throughout the captions of the Supplementary Figures.

Triptolide is mentioned only once, in the figure legend. I don't know this compound, but based on the context I guess it is inhibiting RNA transcription. In the literature, it seems very toxic to cells. What is it supposed to be doing? Where did they buy it? What is the cellular IC50 of triptolide and what concentration was used in these assays?

Triptolide binds to the XPB subunit of TFIIH. The ATP-dependent helicase activity of XPB is required for the first step in transcription to open the double-strand DNA and create a "transcription bubble". Triptolide inhibits the ATPase activity of XPB, thus it prevents the formation of the "transcription bubble" and hence initiation of transcription. Triptolide treatment is irreversible as it binds covalently to XPB and induces a fast proteasome-dependent degradation of RNAPII.

In the revised version of the manuscript, we specified in the *Main Text* as well as the caption of Fig. 1 that triptolide is a transcription inhibitor together with a pertinent reference, i.e. (Bensaude (2011) *Transcription* 2(3):103-108).

Are the differences in fig 1C and S3B statistically significant? In S3B, it appears n=1 in many cases. Can the authors justify why they are showing n=1 data (e.g. for NR1D1, PER1, PER2?).

In the revised version of the manuscript we added the corresponding statistical analysis to Fig. 1C *bottom* and Fig. S3B and specified the P values determined from a t-test in the caption of the figures.

The experiments underlying Fig. 1C and Fig. S3B successively expanded throughout this study. Thus, it happened that for some clock genes less individual experiments have been executed. However, it should be noted that each data point in Fig. 1C *bottom* and Fig. S3B stems from an individual experiment where the results from three technical replicates have been averaged. We have become aware that this was not properly reported in the original version of the manuscript and, thus, now specified these details in the captions of Fig. 1 and Fig. S3.

Is the increased stability of mRNA of multiple core clock genes after CPSF6 knockdown (Fig S3) responsible for the longer period? Certainly tau, which decreases stability of PERs, decreases period, and increased stability of PER2, increased period length.

In our study the stability is only (non-significantly) increased in case of *CRY1* for *CPSF6* depleted cells which is not accompanied by an alteration of the total mRNA expression nor a change in polyadenylation site selection, see Fig. S3 B. Thus, an increased stability of mRNA in core clock genes is unlikely as a cause for

the longer period. However, in our response to Comment 2 of Reviewer 3 we further discuss examples of clock genes having a shorter half-life, i.e. a decreased stability after knockdown of *CPSF6*.

In addition, we realized that the observation of this reviewer that “multiple core clock genes show an increased stability” might stem from the reverse ordering of subpanels in Fig S3B compared to the corresponding ordering in Fig. 1C *bottom*. We sincerely apologize for this flaw and rearranged the subpanel ordering of Fig. S3B in the revised version of the manuscript.

What are the units shown in Table S8? (note I didn't go through all the tables, but I think all were pretty sparse in explanations).

In the revised version of the manuscript, we substantially expanded the table captions to describe the content of the Tables in more detail.

Reviewer #3: In chronobiology the regulation of circadian period length and its temperature compensation remain major unsolved issues. The authors performed a screen in U2OS cells for genes involved in post-transcriptional regulation and RNA processing which affect period length and temperature compensations. Out of 1024 genes, they identified that CPSF6 knockdown lengthens the period, and then showed that the compensation is also reduced (Q10 increased to 1.19 in U2OS). As CPSF6 is involved in 3'-end processing of transcripts and polyadenylation (PA) site selection the authors then combined 3-end transcriptomics and proteomics to identify the mechanism leading to the period and compensation phenotype wild-type and CPSF6 knockdown cells. While the knockdown induced many differential change in PA sites, mRNA and protein expression, the changes found upon different ambiant temperatures were largely independent of CPSF6. An integrative analysis of all the datasets to select for candidate genes that fulfill the characteristics of a regulator of temperature compensation led to the identification and testing (siRNA) of EIF2S1. Overall this is a well executed study with a large amount of new datasets, which overall sheds light on the complex CPSF6 biology, and how CPSF6 is implicated in period temperature compensation, possibly through EIF2S1 (aka eIF2α). While temperature compensation for the circadian period is usually thought to stem from the properties of core clock proteins, it is a new concept that global regulators such as CPS6 might also play a major role. Therefore the finding are novel and will be of great interest for PLoS Biology readers.

We gratefully thank the reviewer for the kind words and acknowledging the novelty and interest of our study. The detailed suggestions as well as the proposed additional analysis substantially improved the manuscript.

Comments

1. Fig S1. It is not quite clear where the dose-dependent lengthening is shown. This may be an issue of the caption or axes labels (do the labels refer to the three constructs or doses?). Also the authors mention that period is similarly lengthened in 3T3 cells, which is important, but those results seems not as clear with respect to the doses? Do the authors have data from 3T3 cells that recapitulate the temperature compensation in U2OS in Fig. 1B? Since this is the key experiments that links with temperature compensation, this would be important to show.

By "dose-dependent lengthening" we wanted to express that the strength of the period lengthening phenotype depends on the efficiency of CPSF6 knockdown, i.e. the circadian period correlates with the *CPSF6* mRNA expression. In the revised version of the manuscript we reformulated the corresponding paragraph to make this clearer. In addition, we re-ordered the sub-headings and captions.

Unfortunately, we do not have data from 3T3 cells and due to limited resources we have not been able to investigate this issue. Instead of providing data for 3T3 cells, in the revised version of the manuscript, we studied to which extend the period lengthening phenotype of *NUDT21* knockdown (also known as *CPSF5*) is temperature sensitive. New Supplementary Figure S2 B shows that a knock down of *NUDT21* phenocopies the effect of *CPSF6* depletion, giving further evidence that *CPSF6* indeed acts on the clock via its role in the polyadenylation complex.

2. Are the shorter half-lives and lower expression levels of the reported clock genes (Clock, Nr1d2) consistent with longer period? I.e. is the partial knockdown of those genes giving the same phenotype (see for example Baggs PLoS Biology 2009)? Or perhaps the authors have their own data on this?

The short half-life and lower expression level of *CLOCK* in *CPSF6* depleted cells (Fig. 1C) is consistent with a long period phenotype with respect to previous studies, reporting that homozygous *Clock* mutant mice have a long period or sometimes arrhythmic circadian phenotype under constant darkness at the behavioral level, see e.g. (Vitaterna et al. (1994) *Science* 264: 719-725) or (Ochi et al. (2003) *Neuroscience Letters* 347: 163-166). In (Baggs et al. (2009) PLoS Biology 7(3):e1000052)

mentioned by the Reviewer, shRNA mediated knockdown of *CLOCK* in U-2 OS cells leads to an arrhythmic phenotype.

Similarly, in Fig. 1 of (Baggs et al. (2009) PLoS Biology 7(3):e1000052)) a long period circadian phenotype after *NR1D2* (also known as *REV-ERBβ*) depletion can be observed, being consistent with the long period phenotype, a shorter *NR1D2* mRNA half-life and lower expression after *CPSF6* depletion (Fig. 1B,C). Further (computational) evidence for a longer period upon reduced *NR1D2* half-life can be obtained by revisiting a previously published mathematical model of the mammalian core clock that contains the concentration of *REVERBs* as a dynamical variable (van Soest, Olmo, Schmal, Herzel (2020) *J. R. Soc. Interface* 17: 20200556). A bifurcation analysis of this model, i.e. computationally tracked changes in the qualitative and quantitative behavior of the model upon parameter changes – here the *NR1D2* half-life, reveal that a reduction in *NR1D2* half-life (i.e. moving left from the nominal model parameter as depicted by a *vertical gray dashed line*) leads to a reduction in amplitude, baseline expression and lengthening period, at least in the (larger) vicinity of the nominal parameter set.

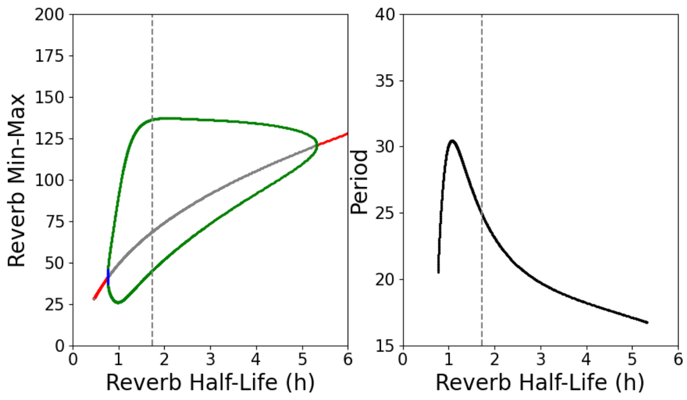

3. Half-lives measurements. To which isoforms do the measurements refer to? Is there a way to distinguish and would this matter for the potential mechanism of period compensation?

We thank the reviewer for this comment. In the revised version of the manuscript we extended the caption of Supplementary Figure S3 in order to provide more details. Primer locations that are used to measure total and long 3′ UTR expression levels are depicted by green arrows. In addition, NCBI reference IDs together with isoform length in base pairs and the gene symbol are given.

4. Overall the authors have not used the length differences of the distal/proximal 3'UTR isoforms in their analysis. Based on the function of the polyadenylation complex, one would expect to see dependencies with length in several variables (%usage, etc).

In addition to analyzing the normalized center of read distributions (NCOMs) along the 3′ UTRs we now appended an analysis that takes into account the length of the isoforms. New

Figures S5 C and D show results for the non-normalized read distributions which we termed “COMs” in analogy to our previous notation. By this we quantified that, globally, the median COM is reduced by 287 nucleotides in *CPSF6* depleted cells compared to the control. We have extended the corresponding text in the second paragraph of *Section 2.2*.

5. Could the authors elaborate further on the relation between mRNA and protein levels? Is this global correlation, or lack of correlation in certain cases, excepted and comparable to other datasets? What are the reasons? Are there certain classes of proteins that show more, or less correlation, etc.

In the manuscript we inferred a high correlation between transcript and protein abundance from the fact that proteins which have a significantly different abundance in *CPSF6* depleted compared to wild type cells are enriched (Fisher’s exact test) in the group of genes showing a significant regulation at the transcript level. We further probed this correlation by directly comparing the transcript and protein abundance. A highly significant correlation with a Pearson correlation coefficient of r=0.43 (p<<0.01, log-log scale, *gray dots* in below figure’s upper left panel) that further increases for genes that are significantly altered at both levels (cyan dots) can be observed. This correlation is, e.g., slightly smaller than the value of r=0.64 for the mRNA versus protein copies per cell (log-log scale) reported in (Schwanhäusser et al. (2011) Nature 473:337-342) that we cite in the main text.

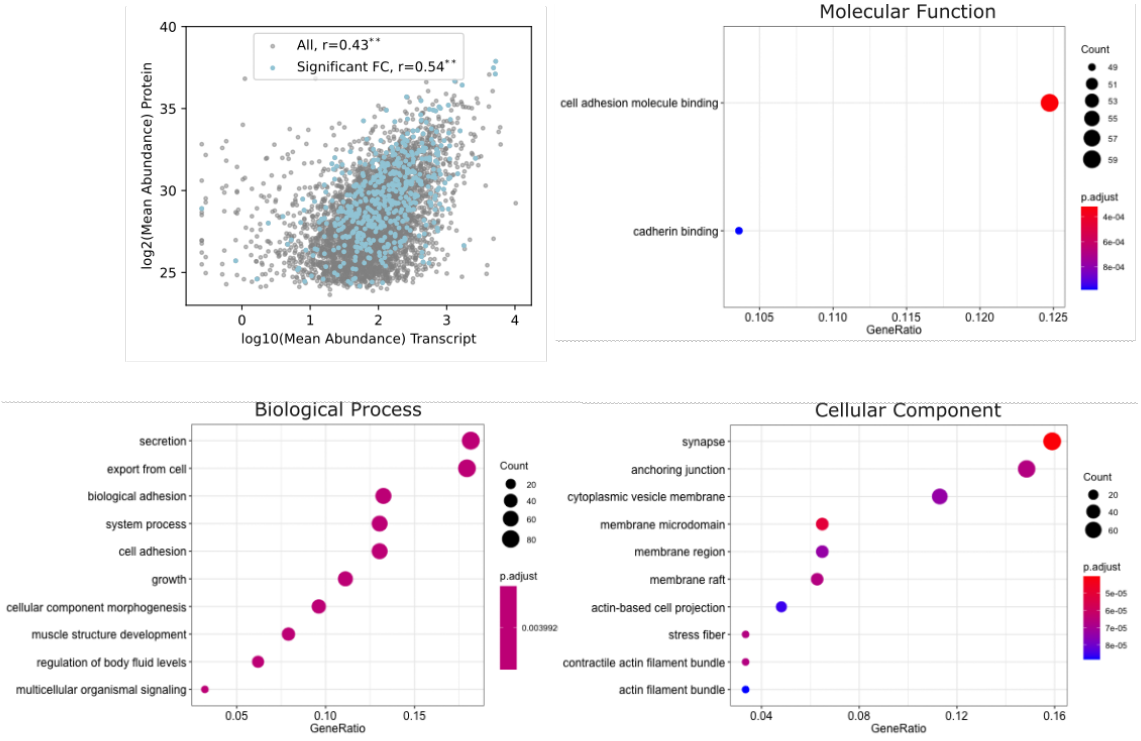

In order to search for classes of proteins that show a high correlation between the fold changes at the protein and transcript level upon *CPSF6* knockdown we applied a gene enrichment analysis using the enrichGO function of the R package clusterProfiler (Yu et al. (2012) OMICS 16:284-287), see upper right and bottom panels in above Figure.

Minor
In general, is it typical that period mutants also show temperature compensation defects? The authors might discuss some striking examples.

We gratefully thank the reviewer for this suggestion. In the revised version of the manuscript we substantially extended section *Introduction* to introduce previously published temperature compensation phenotypes such as the *tau* mutation in hamster and the per^L mutation in the fruit fly *Drosophila melanogaster*. Both mutants have been originally described as circadian clock mutants affecting the period length before the role of the mutation in temperature compensation has been discussed. Thus, it is known that several period mutants show a temperature de-compensation phenotype.

However, not every period mutant shows a temperature de-compensation phenotype as shown, e.g., for *Cry1* or *Cry2* deficient clocks in mouse ESCs derived from $PER2^{Luc}$ knock-in mice showing a period shortening or lengthening phenotype, respectively, but no alteration in temperature compensation (Tsuchiya et al. (2016) *J. Biol. Rhythms* 31(1): 48-56). See also the exhaustive list of circadian rhythm mutants in *Neurospora crassa* categorized into those exhibiting temperature compensation and those that loose their temperature compensation capacity in Table 1 of (Bell-Pederson (2000) *Fungal Genetics and Biology* 29: 1-18).

We shortly summarized this finding in the revised version of the third paragraph in section Introduction and added the references (Tsuchiya et al. (2016) *J. Biol. Rhythms* 31(1): 48-56) as well as (Bell-Pederson (2000) *Fungal Genetics and Biology* 29: 1-18).

Fig 1C: First and second rows. Are the changes in relative mRNA expression consistent with the total mRNA expression bars (lower row, middle panel)?

The relative mRNA expressions shown in the middle row of Fig 1C are consistent with the total mRNA expression bars in Fig. 1C bottom, middle panel. Here, each data point, overdubbed over the total mRNA expression bars, show the data of one individual experiment where the results of three technical replicates have been averaged. In the relative mRNA expression plots (Fig. 1C middle row), the dynamics of all three technical replicates

within one individual experiment are shown per condition (wild type vs *CPSF6* knockout cells), i.e. representative data underlying one out of the three data points shown in the total mRNA expression bars is depicted.

We now more thoroughly specify these details in the corresponding captions of Fig. 1C and Fig. S3.

"our data suggest that processes other than mRNA stability regulate changes of PER3 expression." Could this be true also for the other clock genes, when consider the quantitative changes of half-lives sand expression levels?

Other clock genes (*ARNTL, CRY1/2, NR1D1, PER1/2*) as depicted in Fig. S3B generally neither show a large shift in polyadenylation site usage nor an alteration in mRNA half-life upon *CPSF6* knockdown. The only exception is *CRY1* that shows a mild (but non-significant) increase in mRNA half-life that is accompanied by a slight increase in mRNA expression, being consistent with a stability-induced expression change. One can observe an increase in mRNA expression upon *CPSF6* depletion for *ARNTL*, *NR1D1*, *PER1* and *PER2* even though no change in mRNA half-life has been detected. In a tightly connected regulatory network of interlocked feedback loops such as the circadian clock, the observed increases in clock gene expression could well be due to the effect of *CPSF6* knockdown on other clock elements.

It is unclear if normalizing the NCOM to the length of the 3' UTR is always a good thing as it might hide some length dependent effects which might be expected.

We reanalyzed our data set, using the non-normalized read distribution (COM) instead of the normalized values, thus taking into account the length of the isoforms. In addition to the new supplementary Figures S5 C and D that depict the shift in COM values upon CPSF6 knockdown, we found that our results regarding the differential temperature response between control and *CPSF6* depleted cells depend little on whether NCOM or COM values have been used (compare Venn diagram below with Fig. 5D of the *Main Text*). The same set of 6 genes showing a differential response at all three investigated layers emerge:

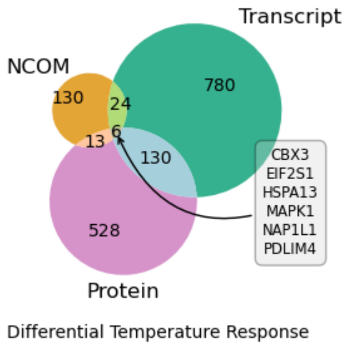

Fig. 2B. Scatter plot of one vs other might be useful as well.

In the new Supplementary Figure S5 we added new subpanel B that depicts a scatterplot of results shown in Fig. 2B as suggested.

In https://pubmed.ncbi.nlm.nih.gov/33648552/ "CPSF6 links alternative polyadenylation to metabolism adaption in hepatocellular carcinoma progression.", there is also a RNA-seq mapping of PASs upon knockdown. The authors should refer to it.

In the revised version of the manuscript, we referenced this work in the second paragraph of section 2.2
