# Supplementary material for: Alternative polyadenylation factor CPSF6 regulates temperature compensation of the mammalian circadian clock
Source: PLoS Biol. 2023 Jun 28;21(6):e3002164. doi: 10.1371/journal.pbio.3002164 (PMC10335657; doi:10.1371/journal.pbio.3002164)
Supplement: Attachment — Submitted filename: Response_to_Reviewers.pdf [file pbio.3002164.s034.pdf]

Response to Reviewers and Editors

Editorial comments:

The reviews are appended below and as you will see the reviewers appreciate the substantial effort that has gone into this revision and Reviewers 2 and 3 have suggested we accept the study. After careful discussion with the Academic Editor, we think that the current revision has addressed the reviewer comments to our satisfaction and were are therefore likely to accept the study. However, we note that Reviewer 1 has a number of lingering concerns and additional points that may require further consideration. We therefore wanted to give you the chance to read and respond to Reviewer 1's remaining reservations, and we encourage you to adopt any changes to the manuscript related to these points that you think would improve the study.

We gratefully thank the Editor and the Academic Editor. With our detailed responses below we hope to have addressed all of the remaining concerns of Reviewer 1.

**IMPORTANT: Additionally, before we can accept your manuscript for publication, we have a number of editorial requests which we need you to address:

1) BLURB: Please provide a blurb which (if accepted) will be included in our weekly and monthly Electronic Table of Contents, sent out to readers of PLOS Biology, and may be used to promote your article in social media. The blurb should be about 30-40 words long and is subject to editorial changes. It should, without exaggeration, entice people to read your manuscript. It should not be redundant with the title and should not contain acronyms or abbreviations.

Done.

2) DATA REQUEST: Thank you for uploading your sequencing and proteomic data to data repositories. I tried to access the proteomic dataset on the ProteomeXchange Consortium via the PRIDE (https://www.ebi.ac.uk/pride/archive ; username: reviewer_pxd029343@eb i.ac.uk and password: bOWYvyOk), however I did not see the dataset (PXD029343). Can you please double check that the information to access this dataset in your data availability statement is correct?

We double checked and were able to access the data on the PRIDE Archive.

a) Please go to https://www.ebi.ac.uk/pride/archive

b) Click on "Log In"

c) Use reviewer_pxd029343@ebi.ac.uk as username and bOWYvyOk as password.

In case of acceptance, we will make the proteomics as well as transcriptomics data publicly available.

3) DATA REQUEST: You may be aware of the PLOS Data Policy, which requires that all data be made available without restriction: http://journals.plos.org/plosbiology/s/data-availability. For more information, please also see this editorial: http://dx.doi.org/10.1371/journal.pbio.1001797

While the deposition of the sequencing and proteomic datasets meets this requirements for most of your figure panels - for any figures not related to those datasets, we need you to provide the underlying data. (maybe Fig 1B_C; Fig S1, S2, S11; Supp text figure 4).

Note that we do not require all raw data. Rather, we ask that all individual quantitative observations that underlie the data summarized in the figures and results of your paper be made available in one of the following forms:

a - Supplementary files (e.g., excel). Please ensure that all data files are uploaded as 'Supporting Information' and are invariably referred to (in the manuscript, figure legends, and the Description field when uploading your files) using the following format verbatim: S1 Data, S2 Data, etc. Multiple panels of a single or even several figures can be included as multiple sheets in one excel file that is saved using exactly the following convention: S1_Data.xlsx (using an underscore).

b - Deposition in a publicly available repository. Please also provide the accession code or a reviewer link so that we may view your data before publication.

Regardless of the method selected, please ensure that you provide the individual numerical values that underlie the summary data displayed in the following figure panels as they are essential for readers to assess your analysis and to reproduce it.

NOTE: the numerical data provided should include all replicates AND the way in which the plotted mean and errors were derived (it should not present only the mean/average values).

Please also ensure that figure legends in your manuscript include information on where the underlying data can be found, and ensure your supplemental data file/s has a legend. For example, to each figure legend (including supplemental) you can add the sentence "the data underlying this figure is available from the NCBI Gene Expression Omnibus (GEO; https://www.ncbi.nlm.nih.gov/geo/) under accession number GSE185896, the ProteomeXchange Consortium via the PRIDE partner repository with the dataset identifier PXD029343, or is contained in S1_Data."

We deposited the underlying data within the manuscript as Supplementary data files and modified the captions of the Figures accordingly.

4) GELS AND BLOT REPORTING: We require the original, uncropped and minimally adjusted images supporting all blot and gel results reported in an article's figures or Supporting Information files. We will require these files before a manuscript can be accepted so please prepare and upload them now. Please carefully read our guidelines for how to prepare and upload this data: https://journals.plos.org/plosbiology/s/figures#loc-blot-and-gel-reporting-requirements

Please provide the uncropped and unadjusted gel related to Supp Text Fig 2 (page gel) - annotated as described in the guidelines above.

The unadjusted gel has been deposited in a corresponding Supplementary Data file.

Published Peer Review History
Please note that you may have the opportunity to make the peer review history publicly available. The record will include editor decision letters (with reviews) and your responses to reviewer comments. If eligible, we will contact you to opt in or out. Please see here for more details: https://blogs.plos.org/plos/2019/05/plos-journals-now-open-for-published-peer-review/

We are happy to make the peer review history publicly available.

Press
Should you, your institution's press office or the journal office choose to press release your paper, please ensure you have opted out of Early Article Posting on the submission form. We ask that you notify us as soon as possible if you or your institution is planning to press release the article.

Ok.

Protocols deposition
To enhance the reproducibility of your results, we recommend that if applicable you deposit your laboratory protocols in protocols.io, where a protocol can be assigned its own identifier (DOI) such that it can be cited independently in the future. Additionally, PLOS ONE offers an option for publishing peer-reviewed Lab Protocol articles, which describe protocols hosted on protocols.io. Read more information on sharing protocols at https://plos.org/protocols?utm_medium=editorial-email&utm_source=authorletters&utm_campaign=protocols

N.A.

Reviewer Comments:

Reviewer #1: The revised manuscript by Schmal et al., is improved and addressed many of my original comments. Because of this, the authors are strongly encouraged to include additional data and text in the response letter to the main manuscript, as some data are integral to support the validity of the study. Below, I describe a few remaining reservations. I also added further thoughts based on new information provided in the revised version. The authors addressed all the other comments.

We thank the reviewer again for the suggestions that we believe substantially improved the paper.

Responses to the major points
1. The authors provided additional data, in which the efficiency of knock-down was evaluated at three different temperatures. The data are an important piece of information to support the authors' conclusion and hence should be added to the manuscript. At the same time, the data, as currently presented, lack critical information: a) what is 100 in Y-axis? Relative to what?, b) how many independent tests? C) what was the amount (or concentration) of shRNAs in reference to Fig. 1A-B, or Fig. S1A-B?

We follow the suggestion of the reviewer and show the data in a new sub-panel of Figure 1 of the main text. The corresponding caption has been changed accordingly.

In addition, we changed the labeling of sub-panels in Fig. 1 to better structure the main text and corresponding references to the sub-panels.

2. As I believe there is no clear threshold for both absolute Q10 values and relative changes (deltaQ10) to define whether the process is temperature compensated or not, I am fine with leaving this point up to the post-publication peer-review by a broader

scientific community. To execute this well, however, the authors should provide all the information available for the readers so that they can make their own judgment calls.

There are many papers that focus on temperature compensation. Some of these papers calculated Q10 values, while most did not (which actually surprised me!). There appears to be a range of absolute and relative Q10 values, including those similar to the values reported in this manuscript, used to claim whether the process is temperature compensated or not. The authors should succinctly but comprehensively summarize the previous findings and compare those with their finding (also see comments below). The authors picked one paper (Huang et al., Science 1995) and calculated the Q10 value, however, it is not justifiable to cite one paper to support the significance of the authors' findings.

Other examples (that I was able to find):
https://doi.org/10.1128/mBio.01425-21
https://www.jstor.org/stable/4267082
https://doi.org/10.3389/fphys.2022.888262
https://doi.org/10.1016/j.isci.2020.101388
https://doi.org/10.1177/0748730415613888
https://doi.org/10.1371/journal.pcbi.1005501
https://doi/10.1126/sciadv.abe8132 (cited but not in this context)
https://doi.org/10.1016/j.molcel.2015.08.022 (cited but not in this context)
https://doi.org/10.1073/pnas.0908733106 (cited but not in this context)

To clarify, I am not requesting the authors calculate Q10 values of all of these studies. Rather, I am requesting the authors provide all the information so that readers can be fully informed to draw their own conclusions.

This is the authors' statement: "circadian free-running periods have been found to be remarkably resilient to changes in ambient temperature, typically exhibiting Q10 temperature coefficients between 0.85 to 1.2 (lines 22-24)". Meanwhile, their major finding in this manuscript is that Q10 values are different between control and Cpsf6 KD (Ctrl: 1.1 vs Cpsf6 KD: 1.2), but both values are within the range of temperature compensation defined by the authors (based on citations). I am simply confused - if there are changes in Q10 values between different conditions, even if the absolute Q10 values are within the range of "temperature compensation (e.g., 0.85-1.2)", should that be considered as temperature de-compensation or under-compensation? What changes are small enough to say that it is NOT temperature compensated if statistics support the difference? Please

summarize current consensus in the field and discuss this further.

We used a statistical argument to define temperature de-compensation. Along these lines, a clock is temperature de-compensated if the slope describing the temperature dependency of the free-running period in the mutant is significantly different from such slope in the relatively temperature-compensated control cells. Thus, temperature de- or under-compensation is a feature relative to the behavior in the respective control cells, tissues or organisms. Q10 values of such controls generally depend and vary between organisms. For example, the Q10 value in the wild type Canton strain of *Drosophila* is 0.98 while it is approx. 1.1 in retina cells of wild type golden hamster (see introduction). In addition, the temperature response of the free-running period can be non-linear as shown for different *Neurospora crassa* wild type and mutant strains, where the behavior could be divided into temperature ranges with different Q10 values, depending on the mutant background, see Fig. 1 of (Gardner et al. (1981) Plant Physiol. 68: 1244-1248).

In order to not confuse the reader and to better account for the variability of Q10 values across previous publications we remove the explicit numerical values 0.85 – 1.2 from the introduction as we give more specific examples later, i.e. for retina cells in golden hamster and the Canton *Drosophila* strain. We slightly revised the corresponding section in the *Introduction* and mentioned the potential non-linear dependency of the free-running period as shown for *Neurospora*, citing the pertinent literature (refs. 19 and 20).

4. (This is also related to the response to the last minor point): The authors mentioned in the response letter that they designed the experiments based on "prior knowledge from previous studies on temperature compensation that found post-transcriptional mechanisms to play a major role in temperature compensation". Please provide references for these studies. I checked the references 35, 36, 37, 38, 39, and 41, and these are either describing thermo-sensitive alternative splicing (not temperature compensation) or roles of casein kinase (post-translational, not post-transcriptional, regulator) in regulating temperature compensation. I agree that thermo-sensitive alternative splicing may be the mechanism to detect the difference in temperature, but I was not able to find any papers demonstrating that alternative splicing (or other pre-mRNA processing processes) is a mechanism underlying temperature compensation. Or post-transcriptional mechanisms playing a 'major' role in

temperature compensation. As the authors are probably aware, changes in period are not the same as temperature compensation (refs 43, 44).

The reviewer is right. In the rebuttal letter we have not been careful enough to point out that our motivation to investigate genes involved in post-transcriptional mechanisms has been based on two observations (1) their role in temperature sensing and (2) temperature compensation.

In the manuscript we more carefully introduced this motivation by always referring to temperature sensing and compensation back-to-back. As the reviewer pointed out, the evidence is stronger for post-transcriptional mechanisms underlying temperature sensing compared to temperature compensation. However, in Diernfellner et al. 2005 (ref 43) the ratio of the long and short isoform of *Neurospora* clock protein FRQ has been suggested to be crucial for temperature compensation which, in addition, can be affected by splice site mutations (see Fig. 3D of ref 43).

Other new points:

Do the authors really think CPSF6 is important for temperature compensation? There are some statements in the manuscript that suggest otherwise (e.g., lines 283-284, 393-398).

Indeed, we present evidence that *CPSF6* is important for temperature compensation as its knock-down significantly alters the temperature-compensation phenotype in U-2 OS. Our finding that knockdown of *CPSF5* but importantly not *CPSF7* phenocopies the effect of *CPSF6*-KD further strengthens the idea that this phenotype depends on the role of CPSF6 within the polyadenylation machinery.

Regarding lines 283-284 and 393-398: As stated in the manuscript, the finding that the cellular temperature response *at the global level* seems to be largely independent of CPSF6 does not exclude the possibility that CPSF6 regulates (one or more) key players underlying temperature compensation via its role within the polyadenylation machinery. Indeed, we find several isoforms showing a differential response of their 3' UTR length with respect to temperature between wild type and *CPSF6* depleted cells (Fig. 5A) but also across more than one regulatory layer (Fig. 5D).

In addition, temperature compensation is one of the main characteristics in defining circadian rhythms observed in almost all living organisms on Earth, while alternative polyadenylation is primarily a mechanism for eukaryotic gene expression.

Even though temperature compensation of free running periods seems to be a fundamental property of circadian clocks among all organisms, convergent evolution might have led to different underlying molecular mechanisms. For example, transcriptional-translational negative feedback loops are generally believed to be a fundamental mechanism underlying circadian rhythm generation at the intracellular level. Nevertheless, circadian rhythms are observed in systems without transcription such as the KaiC phosphorylation cycle that is believed to be a core mechanism underlying circadian rhythm generation in prokaryotic cyanobacteria. It could even be reconstituted in vitro and has been shown to be relatively temperature compensated (Nakajima et al. (2005) Science 308 (5720): 414-415).

The authors also mentioned alternative polyadenylation is highly tissue-specific (lines 437-482). Do the authors think that CPSF6-mediated temperature compensation (if true) is tissue-dependent? Do multicellular organisms have some tissues temperature compensated and other tissues not? How do these organisms exhibit temperature compensation at an organismal level if the regulation is tissue-specific? The authors should reconcile these statements and discuss this further.

Tissue-specific differences in *CPSF6*-mediated temperature compensation is mentioned as a potential future direction of research in the second-to-last paragraph of the Discussion. Being part of an "outlook" paragraph in the Discussion we have no data on this topic yet, but simply mention that both circadian clock properties and alternative polyadenylation are tissue specific and could thus both contribute to a tissue-specific fine-tuning. As for circadian rhythms (where properties in different cells, tissues and organs vary), temperature compensation at the organismal level could be a feature that arises as an emergent network level property through coupled dynamics from single cell, to tissue and organ interactions that collectively respond to environmental stimuli such as temperature.

Along the same line, the authors should also discuss how their new findings align with other mechanisms that have been already proposed (i.e., CK-mediated regulation). Do the authors think that either or both is correct or incorrect? Why or why not?

Our data does not question previous data on CK-mediated regulation of temperature compensation. Thus, we believe that both mechanisms (i.e., CK- and CPSF6-mediated regulation) are important for temperature compensated free-running periods of circadian clocks in mammals.

Very minor:

Lines 378-379: There is no data showing that the KD of CSPF6 leads to "a significantly altered temperature compensation" in NIH3T3 cells. Changes in period at one temperature is not the same as the system being temperature compensated.

We gratefully thank the reviewer. Indeed, we wanted to say that knockdown of *CPSF6* leads to a period lengthening phenotype in U-2 OS and NIH3T3 and to a temperature de-compensation phenotype in U-2 OS.

We re-formulated this sentence in the revised version of the manuscript to better account for this.

Reviewer #2: The manuscript is improved by the many changes. The core observation, that CPSF6 regulates the core circadian clock, remains robust. This revision, which clearly required substantial additional time and effort, unfortunately was not able to successfully address additional mechanistic questions raised by this interesting observation.

We gratefully thank the reviewer and hope to be able to address more mechanistic details in future work.

Reviewer #3, Felix Naef (note, reviewer 3 has signed this review): The authors have provided highly thorough and convincing answers to all of my points. Thank you for adding new panels to Figure S5. I highly recommend rapid publication in PLoS Biology. Felix Naef

We gratefully thank the reviewer for his kind words.
